# Embedded Conductive Fiber for Pumpless Liquid–Gas Phase Transition Soft Actuation

**DOI:** 10.1021/acsami.5c03424

**Published:** 2025-05-05

**Authors:** Hao Liu, Changchun Wu, Senyuan Lin, Yunquan Li, Yang Yang, James Lam, Ning Xi, Yonghua Chen

**Affiliations:** 1 Department of Mechanical Engineering, The University of Hong Kong, 999077, Hong Kong; 2 Shien-Ming Wu School of Intelligent Engineering, South China University of Technology, Guang Zhou 510640, China; 3 School of Automation, Nanjing University of Information Science and Technology, Nanjing 210044, China; 4 Department of Data and Systems Engineering, The University of Hong Kong, 999077, Hong Kong

**Keywords:** liquid−gas phase change, electrothermal actuation, soft robots, grippers, robotic gloves

## Abstract

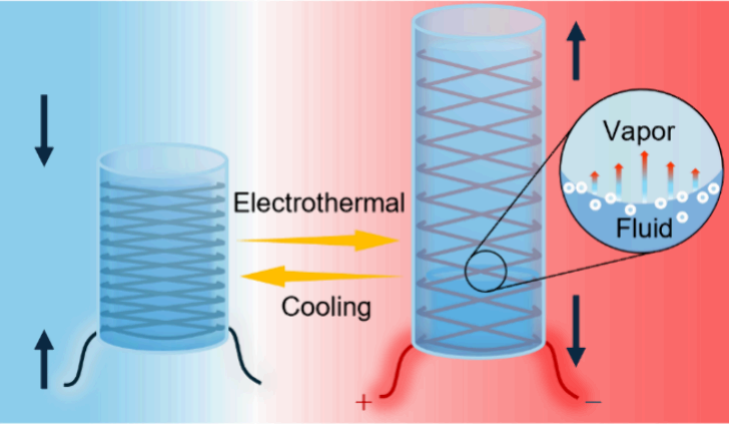

Soft pneumatic actuators are widely used in diverse robotic applications due to their dexterous deformation and conspicuous performance. However, the control and operation of these actuators were restricted by bulky, noisy, and vibrating pneumatic systems. This work introduces a pumpless pneumatic actuator design based on liquid–gas phase transition, named electroconductive fiber-reinforced phase transition actuators (E-FPTAs). Conductive fibers are embedded into the elastomer matrix as flexible heating circuits and morphing programming elements. The E-FPTA generates a high actuation strain of 120% with a low power input of 12 W, showing comparable performance to pump-driven pneumatic actuators. By mechanically programming fiber patterns, the motion type of the E-FPTA can be changed to extending, contracting, twisting, bending, and helical motion, which can be applied for various application scenarios. The E-FPTA is integrated into an octopus-inspired soft gripper and demonstrates multimode grasping in diverse objects. A pumpless robotic glove with eight independent finger joint motions without any pneumatic components is also prototyped. The E-FPTA combines the large deformation of soft pneumatic actuators and the concise structures of the electroactive polymer actuator, which provides a design insight for soft actuations.

## 1 Introduction

Soft actuators exhibit remarkable morphing behaviors, including extending, bending, twisting, contracting, or their combinations.^1^ The unique deformation capabilities have enabled their widespread use in diverse complex applications, such as soft manipulators,^2^ locomotion robots,^3^ biomimetic robots,^4^ and biomedical devices.^5^ Scientists have developed various soft actuators based on advanced materials and ingenious structures, such as pneumatic/hydraulic actuators,^6^ shape memory polymers (SMPs),^7^ dielectric elastomer actuators (DEAs),^8^ ionic polymer metal composites (IPMCs),^9^ hydrogels,^10^ liquid crystal polymers,^11−13^ hydraulically amplified self-healing electrostatic actuators (HASEL),^14^ which could be triggered by fluidic pressure,^15^ electric,^16^ thermal,^17^ light,^18^ magnetic fields,^19^ humidity,^20^ chemical,^21^ or even hybrid stimulus.^22^

Among these, electroactive polymer (EAP) actuators are particularly notable for their ability to deform in response to electrical fields. However, EAPs face inherent trade-offs between low-voltage operation, large deformation, and fast response, which limit their broader adoption.^23^ On the other hand, soft pneumatic actuators (SPAs) are highly favored due to their robustness, safety, economy, easy fabrication, and impressive mechanical performance. To cater to diverse application scenarios, researchers have proposed various SPAs, such as McKibben artificial muscles,^24^ PneuNet actuators,^25^ Origami-inspired actuators,^26^ fabric-based actuators,^27^ flat tube actuators, etc.^28^ Among these, fiber-reinforced soft actuators consisting of an elastomer chamber embedded with nonstretchable fibers show high design degrees of freedom (DOFs).^29^ During inflation, fibers would constrain the chamber expansion. By programming the fiber pattern, the expansion deformation can be transferred to extending, contracting, twisting, bending, or even helical deformation.^30^

Despite their advantages, SPAs are fundamentally limited by bulky, noisy, vibrative, and tethered traditional pneumatic systems. To overcome these challenges, researchers have focused on alternative methods to regulate fluidic pressure without conventional pumps. One effective approach is to compress and transmit the fluid from a reservoir to the SPAs in a closed-loop system, such as the twisting tube actuation (TTA) and compressing bellow actuation (CBA) systems.^31,32^ The TTA and CBA systems improve the controllability of SPAs but also increase mechanical complexity. Another approach involves using chemical reactions, such as fuel combustions and gas evolution reactions (e.g., decomposition of hydrogen peroxide).^33,34^ Although these reactions could generate a drastic pressure ascent, they require continuous replenishment of reactants. Besides, the emission and leakage of reactants and products are potential hazards for humans and the environment.

Liquid–gas phase transition is a reversible process for pressure regulation.^35^ In recent years, researchers used thermal,^36^ light,^37,38^ ultrasound,^39^ and magnetic fields to control untethered soft robots based on phase change materials.^40^ Due to the low energy efficiency of these remote actuation strategies, the application of the untethered phase change actuator is restricted to small-scale and slow response (>1 min) applications. Except for these untethered designs, researchers used coil to stimulate the ferromagnetic particles in the fluid based on induction heating to demonstrate real-world applications like robotic grippers, but an external rigid coil and high-power supply (>100 W) are required.^41^ Embedding the heating circuit into the actuator chamber can effectively improve the heating efficiency,^42,43^ but interferes with the actuator deformation.

**Figure 1:**
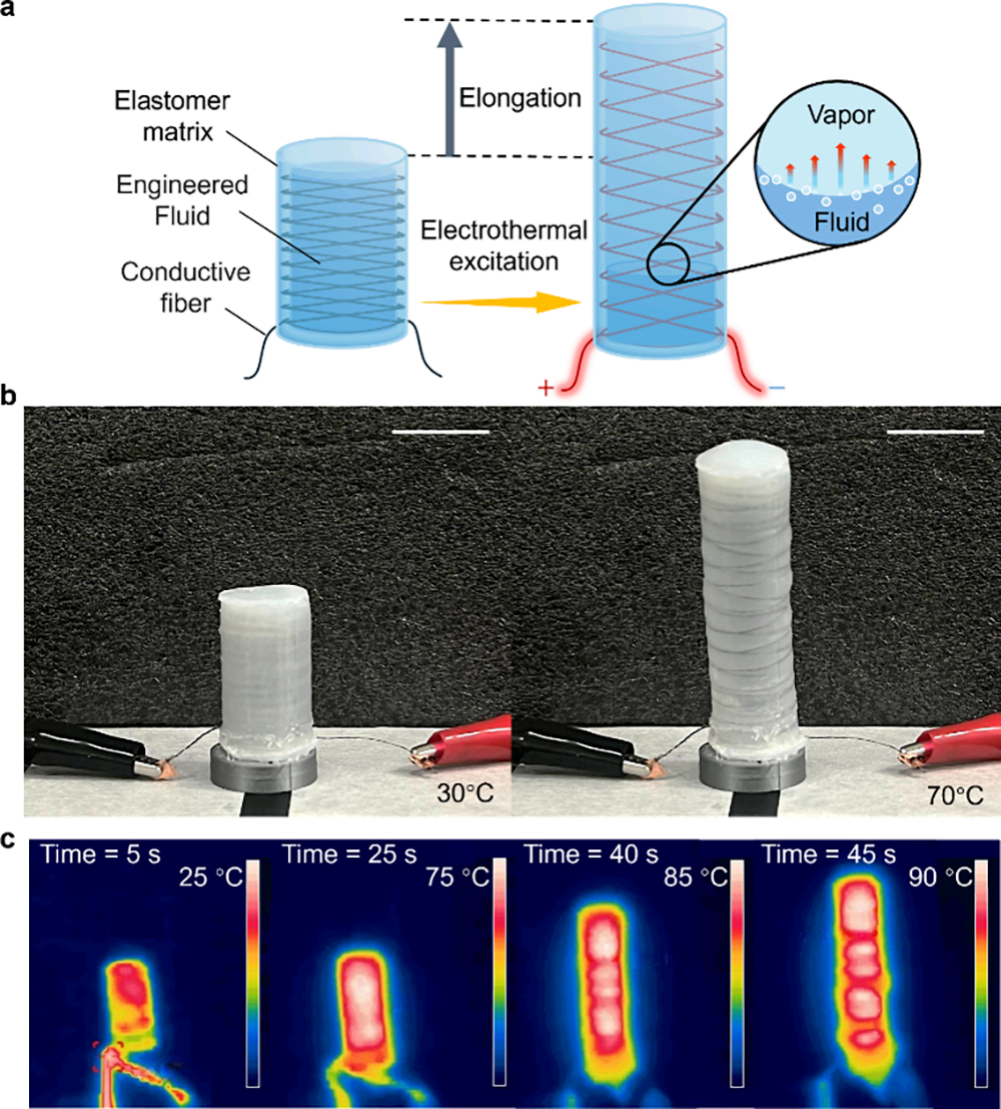
Concept of the electro-conductive fiber-reinforced phase transition actuators. (a) Mechanism of the E-FPTA. (b) Elongation of the E-FPTA prototype at 70 °C. Scale bar, 20 mm. (c) Infrared images of the E-FPTA under different temperatures.

In this study, we proposed novel electro-conductive fiber-reinforced phase transition actuators (E-FPTA) integrating conductive fiber into an elastomer chamber as a flexible heating circuit and deformation constraining element simultaneously without any additional accessories, as shown in Figure 1. By applying low voltage (less than 20 V) to the conductive fiber, the low boiling point liquid will evaporate to increase pressure and deform the soft actuator. The E-FPTA combines the advantages of the large deformation of SPAs and the compact structure of EAPs within a safe voltage input. Additionally, the response speed of the E-FPTA can be easily adjusted by changing the power supply. By mechanically programming the fiber pattern, the E-FPTA can generate extending, contracting, twisting, bending, and helical motions. To demonstrate the potential applications of the E-FPTA, an octopus-inspired gripper is designed for multimode grasping. Furthermore, a pumpless pneumatic robotic glove is proposed with 8-DOFs single joint actuation capability without any pneumatic valves.

## 2 Results and Discussion

### 2.1 Mechanisms of the E-FPTA

Figure 1a presents the mechanism of the E-FPTA, and the deformation of F-FPTA is decided by the braiding pattern of the constraining fiber.^29^ Considering the requirements of high mechanical strength and stable Joule heating, stainless steel fiber is an available choice as a flexible heating circuit and deformation constraining element. By applying electric stimulus on the conductive fiber, the temperature of the metallic fiber increases dramatically, and then transfer heat through the silicone rubber to the low-boiling point fluid. With the temperature increase in the actuator chamber, the low-boiling point fluid evaporates, and the pressure increases to deform the soft actuator. The fluid we used is Novec 7100 engineered fluid with a boiling point of 61 °C because of its biocompatibility, environmentally friendly, low boiling point, and easy condensation at room temperature.

Compared to heating the phase change actuator using an external thermal source, the flexible conductive metallic fiber shows high heating efficiency and does not restrict the actuator deformation. Figure 1b and Movie S1 demonstrate the extending type E-FPTA elongates with a strain of almost 100%. Notably, to avoid the inhomogeneous heating caused by short circuits when the adjacent conductive fiber sections contact directly, as shown in Figure S1a, a silicone rubber layer is precoated on the conductive fiber before fabrication (see the fabrication section). Figure S2a demonstrates the conductive metallic fiber coated by silicone rubber, and the scanning electron microscopy (SEM) images illustrate the metallic fiber embedded in the silicone rubber with a two-ply helical yarn structure composed of torsionally coupled steel microfibers (Figure S2b). Figure 1c shows the infrared image of the E-FPTA driven by Joule heating, and the E-FPTA can extend almost 120% of body length within 50 s.

### 2.2 Thermodynamics of the E-FPTA

**Figure 2:**
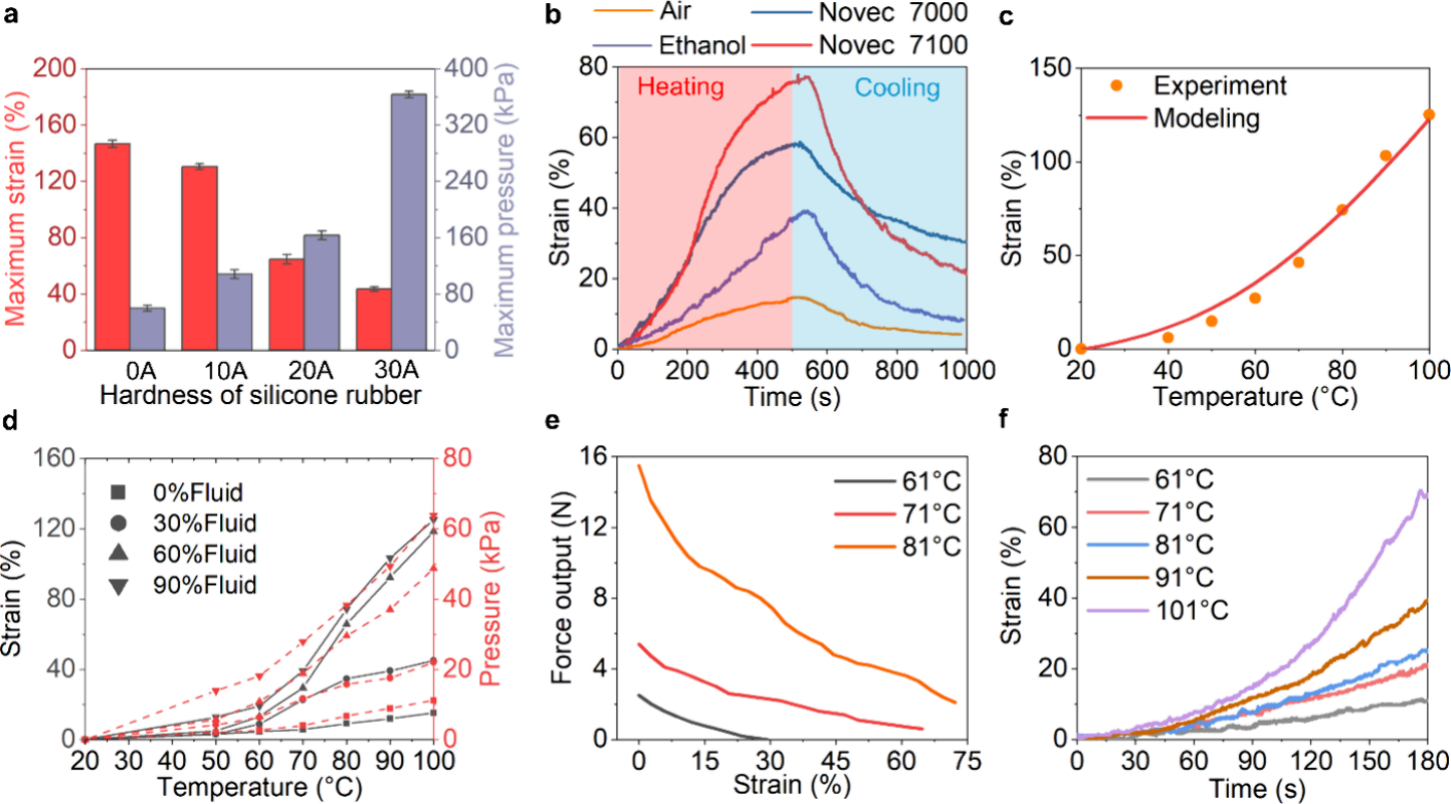
Thermodynamics of the E-FPTA. (a) Maximum strain and pressure requirement of actuators with different silicone rubber hardness. (b) Effect of precharged fluids on strain. (c) Strain of the E-FPTA under different temperatures. (d) Effect of the fluid volume fraction. (e) Illustration of force output versus strain for the E-FPTA excited at 61 °C, 71 °C, and 81 °C. (f) Changes of strain over time under constant temperatures.

To optimize the design of extending type E-FPTA, we investigated the maximum actuator strain of the E-FPTA with diverse silicone rubber hardness (see Figure 2a). The result shows that the actuator made of silicone rubber with less Young’s modulus can generate a large deformation under a small pressure input like the E-FPTA with Shore 0 A silicone rubber can extend almost 145% body length under 60 kPa. The harder silicone rubber exhibits high-pressure resistance and large stiffness, but the strain is also limited. Silicone rubber with hardness Shore 0 A was selected in the E-FPTA design in this study. To determine the proper fluid used in the E-FPTA design, we injected three different low boiling point fluids into the actuators with the same parameters to evaluate the effect of fluid and an actuator filled with air was tested as the control group (see Figure 2b). All fluids were heated to 10 °C above their boiling point, and the control group was heated to 100 °C. Due to the low volume expansion coefficient and high boiling temperatures (79 °C), ethanol is the most inappropriate fluid as a phase change fluid. Novec 7000 engineered fluid can generate high pressure at low temperatures (>34 °C), whereas condensing vapor recovering to fluid is hard due to the high theoretical vapor pressure at room temperature inferred from the Antoine equation (see Figure S3 and Table S1). Because of the easy condensation under room temperature, proper operation temperature, and high pressure output, Novec 7100 is considered the most proper working fluid in the E-FPTA design. We tested the strain of the E-FPTA under different temperatures like Figure 2c, and the E-FPTA shows great actuation performance that extends with a strain of 120% under 100 °C. Analytical modeling is built to predict the actuator strain under different temperature inputs (see eqs 1-18 in the modeling section).

The volume of the fluid injected into actuators is a key parameter affecting actuator performance. The more fluid in the chamber, the larger the strain E-FPTA generates, as shown in Figure 2d. When the 90% chamber is filled with fluid, the E-FPTA can generate 60 kPa pressure and elongate 120% at 100 °C. Figure 2e presents the relationship between the E-FPTA force output and strain in constant temperatures. At 81 °C, the actuator can generate a maximum force output of 16 N. The high force output also verifies the practicability of the E-FPTA. We measured the actuator elongation in constant temperatures from 61 to 101 °C as shown in Figure 2f. The morphing speed improves with the temperature increase and the E-FPTA can extend 70% within 3 min when heated by an external thermal source (see experimental section).

### 2.3 Experimental Characterizations of the E-FPTA

**Figure 3:**
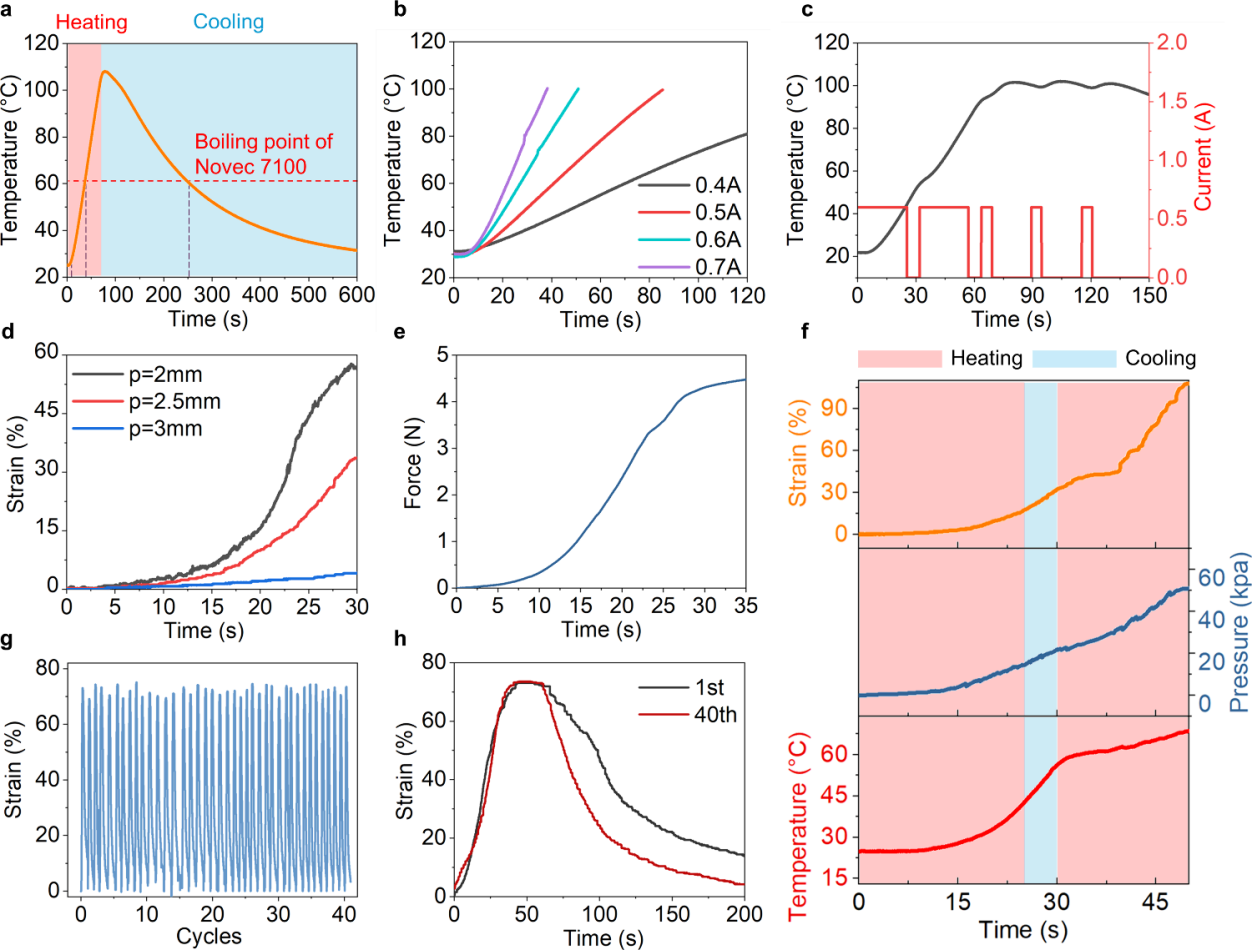
Experimental characterization of the E-FPTA. (a) Temperature rising heated by conductive wire. (b) Effect of stimulus current on temperature change. (c) Duty cycle modulation for temperature control. (d) Effect of the pitch on actuator performance. (e) Force output of the E-FPTA. (f) Illustration of strain, pressure, and temperature of an E-FPTA driven by 0.6 A current. (g) Cyclic test of the E-FPTA. (h) First and last cycles of the cyclic test.

Heating the E-FPTA using embedded conductive fiber is a more efficient way compared with using an external thermal source. For the baseline E-FPTA, the chamber temperature can rise to 61 °C within 35 s, and the power of the heating circuit is less than 12W when the current is 0.6 A (Figure 3a). The most convenient way to enhance the power supply is applying higher current input, and the temperature rise rate increases with larger currents as shown in Figure 3b. However, too large currents will increase the risk of actuator explosion caused by thermal degradation of the silicone rubber (Figure S1b). Because of the limited heat transfer rate, the temperature of the metallic fiber is higher than the silicone rubber matrix. If we desire a constant chamber temperature in EFPTA, the silicone rubber adjacent to the conductive wire will be larger than the target value. So, the duty cycle modulation is required to control and stabilize the chamber temperature to the target value and avoid a too-high temperature gradient between the fiber side and chamber side of the inner rubber cylinder. Figure 3c presents a duty cycle modulation to stabilize the temperature around 100 °C.

The conductive fiber pattern not only influences the actuator’s deformation under pressurization but also decides the temperature regulation of the flexible heating circuit. Using the dense winding pattern, i.e., the less helical pitch of the coiled fiber, will get a larger heating power density and more homogeneous heating. Figure 3d shows that the decrease in the pitch can effectively improve the actuation speed at the same current input (0.6 A). The actuator samples with pitches of 2 mm, 2.5 mm, and 3 mm were stimulated with powers of 17, 14, and 12 W. For the E-FPTA with a pitch of 2 mm, it extends 58% within 30 s, which is far faster than the phase change actuator heating by an external source. The temperature of E-FPTA (p = 2 mm) rises to 100 °C within 38 s (Figure S4). The E-FPTA can exert a pushing force of 4.4 N within 30 s, as depicted in Figure 3e. Figure 3f presents the strain, pressure, and temperature change of an E-FPTA specimen. The power was cut off between 25 and 30 s for duty cycle modulation, and the actuator elongated almost 110% within 50 s. Although the vapor of Novec 7100 will penetrate through the silicone rubber to leak the engineered fluid, reloading the engineered fluid can improve the service life. In addition, the E-FPTA can work continuously for at least 3 h and more than 40 cycles (Figure 3g) without recharging fluid. Figure 3h exhibits the first and last cycles of the cyclic test and does not show obvious performance degradation.

### 2.4 Adjusting E-FPTA Deformation by Varying Fiber Angles

**Figure 4:**
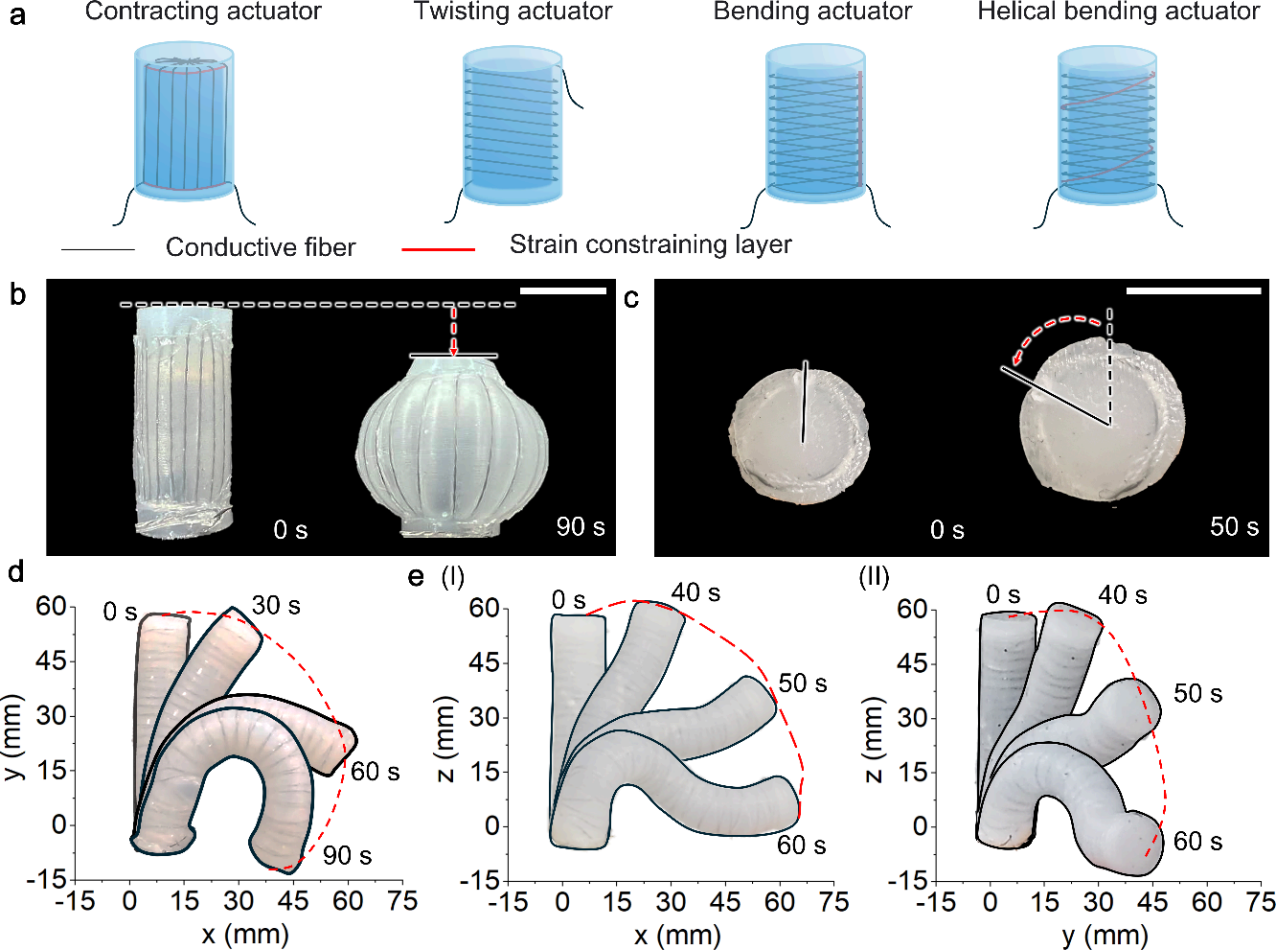
E-FPTAs with programmable fiber patterns. (a) Fiber arrangement of the contracting, twisting, bending, and helical bending actuators. The black line denotes the conductive metallic fiber, and the red line means an insulating strain constraining layer made of Kevlar wire. (b) Prototype of the contracting actuator. Scale bar, 15 mm. (c) Prototype of the twisting actuator. Scale bar, 15 mm. (d) Bending trajectory of the bending actuator. (e) Deformation trajectory of the helical bending actuator. (I) Helical motion in the *x*–*y* plane. (II) Helical motion in the *z*–*y* plane.

By adjusting the fiber pattern embedded in the silicone rubber, the actuator deformation can be programmed to contracting, twisting, bending, and helical bending (see Figure 4a). For the contracting type E-FPTA, the metallic fiber was arranged in parallel with the actuator axis which will buckle to make the actuator contract during inflation. The ends of the metallic fiber in the contracting type E-FPTA are fixed using Kevlar wires for easy fabrication. Figure 4b shows the contracting type E-FPTA when the conductive fiber is parallel to the actuator axis. The actuator contracts 22% at 90 s under a power input of 9 W. For the twisting type E-FPTA, only one helical conductive fiber is wounded on the hollow silicone rubber cylinder to constrain the radial expansion and provide a twisting motion along the helical direction. Figure 4c exhibits a twisting type E-FPTA that could twist 1.73 °/mm within 50 s (5.4 W power). When one side of the extending E-FPTA was fixed using an additional Kevlar strain constraining fiber to restrict the one-sided elongation, the extending motion would be transferred to the bending motion. The E-FTPA bends 3.7 °/mm in Figure 4d, which bends almost 220 degrees at 90 s. Similarly, when an additional constraining fiber was winded helically in the bending type E-FPTA, the actuator will generate a helical bending motion during pressurization like a combination of twisting and bending motion. The motion trajectory of the helical type E-FPTA is shown in Figure 4e. Bending and helical actuators are stimulated with a power of 16.5 W. Movie S2 demonstrates the deformation of the four types of E-FPTA. All parameters of the E-FPTA specimens are introduced in Figure S5 and Table S2.

### 2.5 Octopus-Inspired Soft Gripper

**Figure 5:**
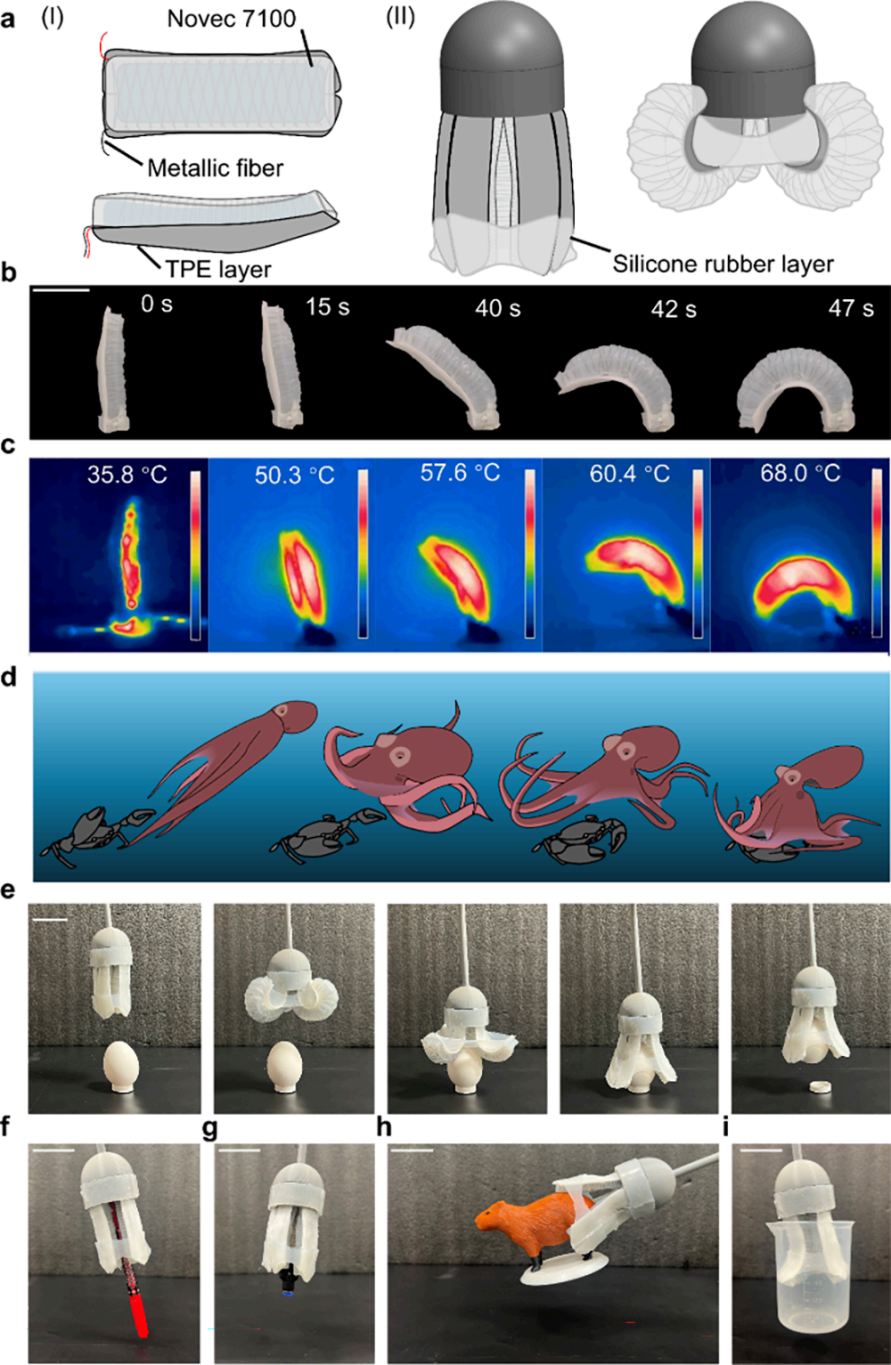
Octopus-inspired robotic gripper. (a) Concept of the soft gripper. (I) Front and side views of the gripper leg. (II) Gripper at initial and actuated states. (b) Actuation of the single gripper leg. (c) Infrared images of the single gripper leg under different bending angles. (d) Predation pattern of the octopus. (e) Grasping of the gripper by mimicking the octopus predation process. (f-i) Multimode grasping of the gripper. (f) Gasping stick-like objects. (g) Grabbing small targets. (h) Capturing prey with bulky size and irregular shapes. (i) Lifting hollow objects. Scale bar, 30 mm.

Octopus tentacles show high DOFs and flexibility due to the support of muscular hydrostats without any rigid bones and joints advancing in the complexity of motions and adaptability. The muscular hydrostats contract anisotropically upon an electric stimulus due to the fibrous architecture.^44^ Inspired by octopus tentacles, we proposed a multimode soft robotic gripper in Figure 5 and Movie S3. The gripper is composed of three bending actuator legs, three pretensioned silicone rubber layer webs, and an outer shell (Figure 5a). The circuit in the shell is demonstrated in Figure S6, and all legs can be controlled by a single power supply. The gripper leg consists of a cuboid E-FPTA filled with Novec 7100 and a 3D-printed leaf-like thermoplastic elastomer (TPE) layer. By applying voltages, the cuboid E-FPTA deforms to bend the TPE layer, and the leg is open. When the power is off, the leg closes under the contraction force of the silicone rubber webs. The TPE layer shows bistable characteristics (see Figure S7), which allows an expansion deformation under a low chamber pressure, and a bending deformation when the pressure surpasses the threshold value (10 kPa). Figure S8 exhibits the relationship between pressure input and bending trajectories of the gripper. The TPE bistable layer enables the actuator to keep bending for a long time in the cooling process and facilitates the alignment between the gripper and grasped objects.

Figure 5b demonstrates the bending trajectory of the gripper leg driven by an electric stimulus. In the beginning, the TPE layer tends to straighten and bend slowly before 40 s. When the actuator bends over the bistable critical state of the leaf-like layer, the bending speeds up and reaches 160 degrees within 47 s. Figure 5c shows the infrared images of the gripper leg in different positions.

The grasping process of the soft gripper mimics the octopus hunting process as shown in Figure 5d. During hunting, the octopus approaches the prey and extends its webs by bending its tentacles outward. And then, the octopus bends its tentacles inside and closes its webs to constrain the motion of targets. For the gripper, three legs close, and the webs shrink into a small circle at the initial state. During grasping, tentacle-like legs bend outward, and webs are stretched and flipped. Shutting off the power supply, legs will be put down to grasp an object, and webs will lock the object. Figure 5e shows the grasping process of the soft gripper, and the gripper can safely lift fragile objects, like an egg.

The soft gripper can grasp objects with diverse shapes using different actuation modes. For the stick-like object, each leg is heated for less than 15 s (like Figure 5b) and the actuators’ chamber expands but the TPE layers morph mildly to squeeze the target (Figure 5f). To save the response time, the leg bends to the critical state enough to grab small objects just like Figure 5g. For targets with bulky sizes and irregular shapes, the gripper should be fully open to capture it (Figure. 5h). Except as a predator, octopuses also hide in shells or stone caverns, and their legs will bend to support and jam themselves firmly on the shelter. The gripper can grasp large objects with hollow structures by mimicking the hiding habit of octopuses like Figure 5i. We also tested the load capability of the soft gripper, and the soft gripper can lift the object with a maximum weight of 500 g (6 times the weight of the soft gripper), see Figure S9.

### 2.6 Soft Rehabilitation Glove

**Figure 6:**
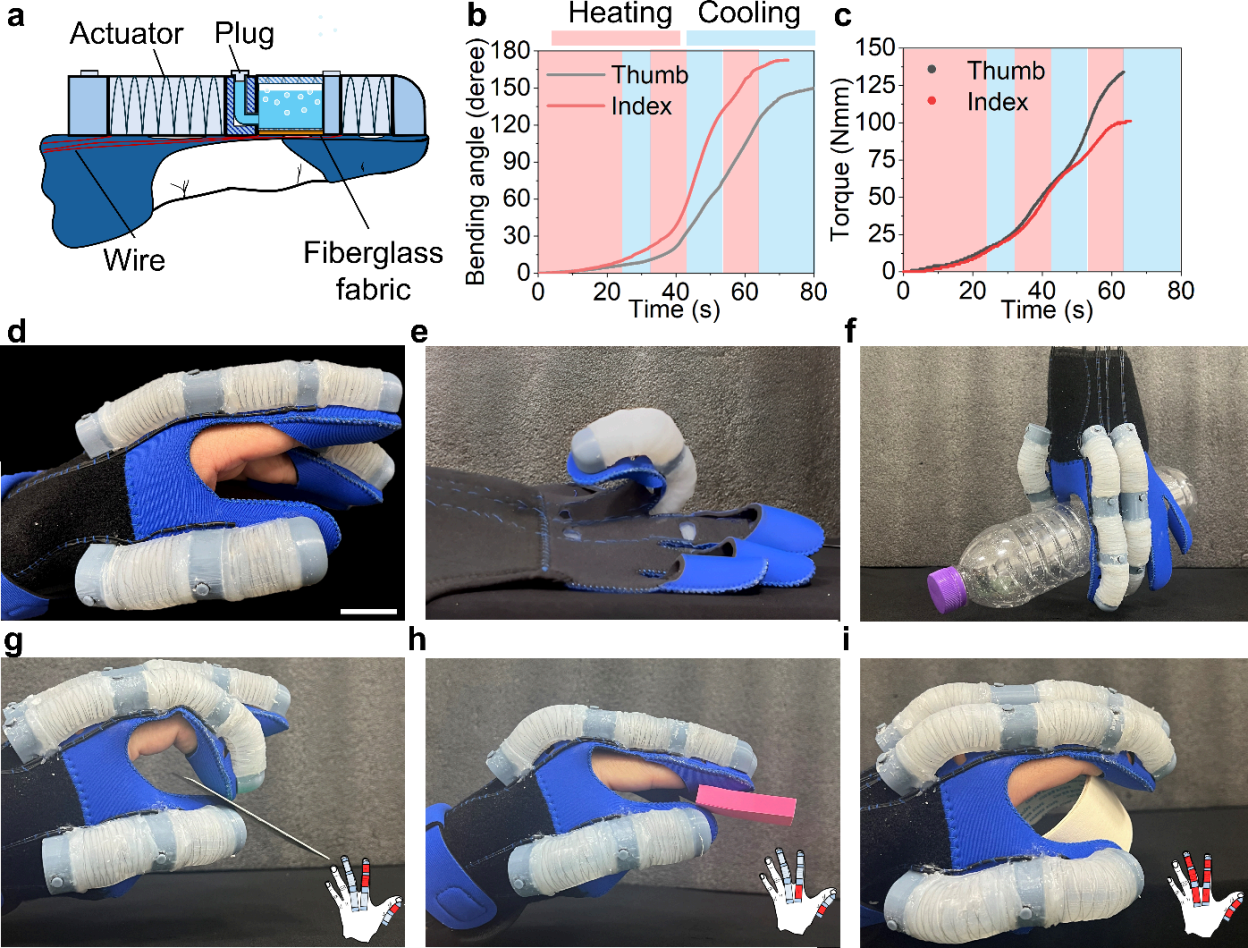
Soft rehabilitation glove based on the E-FPTA. (a) Structures of the robotic glove. (b) Bending angle of the single joint. (c) Torque output of the single joint. (d) Prototype of the robotic glove. Scale bar, 20 mm. (e) Bending trajectory of the index finger. (f) Grasping a bottle with the soft glove. (g) Tip pinch assisted by robotic glove. (h) Palmar pinch assisted by the robotic glove. (i) Power grip assisted by the robotic glove.

People may lose or degrade hand functions caused by diseases, accidents, or even natural aging. Then, soft robotic gloves are essential ways to rehabilitate, assist, and train human hands at home. Among all gloves, the glove based on the pneumatic actuators is the most comfortable, cost-optimal, and effective design.^45^ However, noise, vibration, and bulky and sophisticated pneumatic circuits are always pain points of pneumatic biomedical devices. In this work, we proposed a pumpless robotic glove based on the bending type E-FTPA as shown in Figure 6 and Movie S4. Figure 6a exhibits the structure of the robotic glove, each bending actuator is ergonomically aligned with finger joints to match the motion trajectory of the human hand. The cross-section of bending actuators is semicircular, and the actuator end is connected to a rubber tube for easily reloading and sealing the fluid. The bottom of each actuator is attached to a fiberglass fabric layer for heat insulation and bending motion constraint. Notably, the actuation of each finger joint is independent without valves (Figure S10).

The thumb as the most important finger in human hands has a larger force output compared to other fingers. So, the E-FTA we used in the thumb (radius = 13 mm) has a larger cross-section area compared to other fingers (radius = 10 mm). We tested the bending angle of actuators for the metacarpophalangeal (MCP) joints of the thumb and index finger (Figure 6b) stimulated by a 0.6 A current. The E-FPTA (index finger) can bend almost 160 degrees within 60 s, which meets the requirement of finger joint range of motion. Figure 6c presents the torque output of the two bending actuators, and the glove can provide a torque of 130 N mm in 1 min for the MCP joints of the thumb. The thumb and index finger play vital roles in hand grasping, and a three-finger robotic glove was fabricated for grasping demonstration (Figure 6d).

Figure 6e shows the flexion trajectory of the index finger driven by the robotic glove, and the index finger can easily bend more than 200 degrees. The robotic glove can grasp a plastic bottle like Figure. f. Although the soft glove shows adaptiveness in manipulating unknown tasks, unmatching gestures also cause unnatural sensations for users. For all current robotic gloves, all pneumatic actuators laid on the single finger are connected and share the same pressure supply due to the restriction of the complex valve control. We wore the robotic glove on a human hand and demonstrated the tip pinch, palmar pinch, and power grip assisted by the robotic glove (Figures 6g-i), which shows the advantage of independent control of each finger joint. For the tip pinch, distal interphalangeal (DIP) and proximal interphalangeal (PIP) joints on the index finger and the interphalangeal (IP) joint on the thumb are actuated, as shown in Figure 6g. For the palmar pinch, MCP joints on the index finger and thumb rotate to catch plate objects (Figure 6h). The robotic glove has 8 DOFs and can conduct complex grasping missions benefiting from the pumpless electric E-FPTA.

## 3 Conclusions

This study introduced a novel pumpless soft pneumatic actuation strategy utilizing low boiling point fluid powered by a flexible electrothermal composite consisting of conductive fibers embedded in flexible polymers, which ingeniously serve as the constraining element and heating circuit, named as electro-conductive fiber-reinforced phase transition actuators (E-FPTA). The embedded conductive fiber homogeneously heats the engineered fluid and effectively improves the response speed to less than 1 min which is faster than current actuators based on the liquid–gas phase transition (see Table S4). The E-FPTA shows comparable performance compared with common soft pneumatic actuators and could work silently using an electric power supply without any pumps or motors. The extending type E-FPTA demonstrates a high strain of over 100% and Newton-level force output requiring a low power supply of less than 17 W. The E-FPTA demonstrates substantial deformation under a safe voltage input in contrast to traditional electroactive polymers (EAPs). A comparative analysis of its actuation performance against existing high-performance EAPs is provided in Table S5.^50−54^ Besides, the power of Joule heat from the conductive fiber can be easily controlled by adjusting the electric power input to modulate the actuation speed.

The concise structure makes the E-FPTA desirable for untethered and portable applications because only a battery is required to drive actuators, which broadens the potential application scenarios. In addition, the proposed E-FPTA is scalable and easily customized for other actuation types like contracting, twisting, bending, and helical motions by programming the arrangement of conductive fiber. An octopus-inspired soft gripper based on the E-FPTA is proposed showing multimode grasping for targets with different shapes. The robotic devices based on the E-FPTA show high DOFs without the limitation of the complex pneumatic and electric circuits caused by ponderous valves. A pumpless soft robotic glove with 8 DOFs is fabricated that can achieve complex hand gesture assistance.

There are some limitations of the current E-FPTA that could be explored in future studies. First, the response time of the E-FPTA can be optimized using conductive fibers with stable physical properties and higher power density. The conductive fibers with higher resistance show faster heating speed such as the carbon fiber yarn. Meanwhile, the recovery time at room temperature is still too low. One potential solution is adding nanofillers with high thermal conductivity into the rubber matrix to accelerate heat transfer. Additionally, active cooling can be integrated into the robot system driven by liquid–gas phase change, which is a challenge for future work.^42^ Second, the minimum actuator size is limited to manual fabrication. The soft actuator based on the liquid–gas phase transition can be designed via multimaterial 3D printing techniques using conductive materials as flexible heating circuits, like low melting point alloy or conductive thermoplastic polyurethane, etc.^55^ Finally, the design methods of heating engineered fluid using embedded flexible circuits are also suitable for other soft pneumatic actuators, like PneuNet actuators, Origami-inspired actuators, fabric-based artificial muscles, etc. More E-FPTAs and application scenarios are worth trying in future research.

## 4 Materials and Methods

### 4.1 Materials and Fabrication

All actuators, except for no. 2–4, are made of silicone rubber with hardness shore 0 A. Figure S11 exhibits the detailed process of fabricating the extending type E-FPTA. All molds are made of polyethylene terephthalate glycol (PETG) material using fused deposition modeling (FDM)-based 3D printing technology (A1 mini, Bambu lab, CN). All metallic fibers used in this research are made of 316L stainless steel (0.296 mm diameter, Dongguan Shengxin Special Rope Webbing, CN). The electrical resistance of the metallic fiber is 37 **Ω**/***m***. The fiber is off-the-shelf, low-cost (0.17 USD/m), and high-strength (39.6 N breaking force).

We also tested the joule heating of the conductive nylon wire (1K, Dongguan Shengxin Special Rope Webbing, CN) and carbon fiber yarn (800D, Dongguan Shengxin Special Rope Webbing, CN), as shown in Figure S12. The resistance changes of these conductive fibers and the temperature regulation of the E-FPTAs composed of different fibers are discussed in Figure S13. The resistance of the conductive fibers was measured using a multimeter (DEM12, DELIXI, CN), and each fiber was measured three times to reduce errors.

Before molding the soft actuator, a thin layer of silicone rubber is precoated on the metallic fiber. We first mold the inner silicone rubber layer, wind the fiber, and mold the outer silicone rubber again. After sealing the ends of the actuator, we puncture the bottom of the actuator and insert a silicone rubber tube (4 mm × 2 mm) into the hole. And then glue the tube using an instant glue (XH-2029, Xinhui adhesive, CN). Inject the low boiling point fluid (Novec 7100, 3M, USA) into the chamber using a syringe, and then seal the tube using a PETG plug. The fluid can be easily reloaded via the silicone tube, which is far faster than soaking the actuator in the fluid which requires more than 4 h.^38^

### 4.2 Experimental Setups

#### 4.2.1 Maximum Strain and Pressure of the E-FPTAs

We used E-FPTAs no. 1–4 to measure how the elastomer hardness affects the strain and pressure requirement of the extending actuator. The pressure was supplied by a pressure source and adjusted via a pressure regulator (IR2000–02BG, HIGHEND, FRG). The initial length and maximum lengths of the actuator were measured using a vernier caliper. The tests in Figures 2a, c, d, and e were conducted three times to alleviate the measurement errors.

#### 4.2.2 Strain of the E-FPTA Filled with Different Fluids

For all thermodynamic tests in Figure 2, the actuator was heated using an external thermal source, a flexible silicone rubber heating plate, and EFPTAs used in these tests are the same as No.1. Novec 7100 and Novec 7000 were purchased from 3M. The ethanol we use is medical alcohol (95%). The experiment platform is shown in Figure S14. The elongations of actuators were measured by a laser distance sensor laser distance sensor with a sampling frequency of 100 Hz, and all sensor data in this study were acquired using a data acquisition card (USB-3211, Smacq, CN). For E-FPTAs filled with air, ethanol, Novec 7000, and Novec 7100, the heating plate is adjusted to 100 °C, 89 °C, 44 °C, and 71 °C separately for 5 min, and then turn off the heating circuit to cool actuators under room temperature.

#### 4.2.3 Strain of the E-FPTA under Different Temperatures

The actuator used in Figure 2c is filled with fluid. The volume of the fluid in the chamber can be controlled using the scale on the syringe. The experiment platform is shown in Figure S14a. The E-FPTA was heated from room temperature to 100 °C with an interval of 10 °C. For each interval, the actuator was heating 120 s to a stable state and the actuator length was measured using the laser distance sensor, and the inner pressure was measured using a XGZP6847A air pressure sensor module (0–200 kPa).

#### 4.2.4 Force Output versus Strain of the E-FPTA Under Different Temperatures

The apparatus is shown in Figure S14b, and the actuators are fully filled with fluid. The heating module is the same as the previous experiment. The force output of the actuator was measured using a force gauge (DS2–20N, Zhiqu, CN). Initializing the position of the force gauge, i.e., actuator force output equal to zero. Heat the E-FPTA for 500 s to a certain temperature (61 °C, 71 °C, and 81 °C), and record the force. Then remove the force gauge until there is no obvious force change with an interval of 2 mm for each remove and record the force in each position.

#### 4.2.5 Elongation of the E-FPTA under Constant Temperatures

The setup is the same as that introduced in section 4.2.2, and the actuators are fully filled with NOVEC 7100. The heating plate was preheated to constant temperatures (61–101 °C with a 10 °C interval), and then the E-FPTA was heated for 3 min to record the elongation.

#### 4.2.6 Temperature of the E-FPTA Powered by Conductive Fiber

The temperatures of the inner chamber were measured using a k-type thermocouple sensor (TCM_UA, Dagasensor, CN) in the rest of the tests. The ends of the conductive fiber are connected to an adjustable DC regulated power supply (0–200 V). The E-FPTA no.1 filled with air was energized using 0.4, 0.5, 0.6, and 0.7 A until the temperature of the inner chamber reached 100 °C. Then turn off the power and cool it to room temperature. For the duty cycle modulation, the 0.6 A current through the E-FPTA no.1 filled with air was turned on and off as shown in Figure 2c controlled by a relay module.

#### 4.2.7 Strain of the E-FPTA Powered by Conductive Fiber

For the rest of the tests, all actuators were fully filled with Novec 7100. We tested the actuators with pitch 2 mm, 2.5 mm, and 3 mm to evaluate the effect of pitch on temperature rise and actuator strain. The experiment apparatus is shown in Figure S15. The actuator length was measured using the laser distance sensor. The three actuators were activated by 0.6 A current 25 s and then turned off the power. The pressure and temperature of the actuator can be measured using the sensors mentioned above.

#### 4.2.8 Force Output of the E-FPTA Powered by Conductive Fiber

We tested actuator no.8 using an apparatus similar to Figure S14b. However, the actuator was powered by 0.6 A current 25 s, and the force sensor was fixed.

#### 4.2.9 Cyclic Test

One end of actuator no.8 was fixed, and another end moved linearly along a guide rail like Figure S15. The actuator was powered by 0.6 A current 28 s for each cycle and cooled by a fan to accelerate the recovery process.

#### 4.2.10 Pull-Out Force Test

For the pull-out force test of the gripper, the setup is shown in Figure S9a. The gripper was fixed on a fixture and grasped a 3D-printed PLA cylinder (see Figure S9c). The cylinder was connected to a load cell via a nonstretchable steel wire, and the load cell (5 kg DYLY-106, Bengbu Dayang Sensing System Engineering, CN) was located on a linear sliding table. We measured the force output of the gripper to a distance of 40 mm for each 1 mm interval. We measured the force output under 0 and 10 kPa pressure input controlled by a pressure regulator (IR2000–02BG, HIGHEND, Germany). The pull-out force under each pressure situation was conducted three times.

#### 4.2.11 Uniaxial Tensile Test

The stress–strain curvature of the silicone rubber with hardness shore 0A was measured using the universal testing machine (EZ-LX, SHIMADZU, CN). The silicone rubber specimen is prepared by casting on a 3D-printed PLA mold, and the specimen size is shown in Figure S18.

#### 4.2.12 Scanning Electron Microscopy (SEM)

SEM images were captured by a high-vacuum field-emission scanning electron microscope (ZEISS GeminiSEM 360, Carl Zeiss AG, Germany) with acceleration voltage of 2 kV, and working distance of 7.7 mm.

### 4.3 E-FPTAs with Programmable Deformation

The fabrication of other types of E-FPTA is similar to Figure S11. The parameters of the four F-FPTA are shown in Table S2. For the demonstration in Figure 3, the contracting, twisting, bending and helical type E-FPTAs were stimulated by 0.5 A current with powers of 9, 5.4, 16.5, and 16.5 W, separately.

### 4.4 Soft Gripper

For each gripper leg, the leaf-like layer is made of TPE 83A (TPE filament, esun, CN) using an FDM 3D printer. The parameters of the inner chamber of the cuboid E-FTPA are 22 mm ***×*** 4 mm ***×*** 60 mm, and the outer parameters are 27 mm ***×*** 9 mm ***×*** 65 mm. The metallic fiber was wounded in the middle between the inner chamber and the outer layer with a pitch of 2.3 mm.

For the single leg actuation stimulated by an adjustable DC regulated power supply, the leg was stimulated by 0. Six A current (40 W power input). For the duty cycle modulation, the power turned on 15 s, and then turned off 15 s waiting for heat transfer, and finally heated 8 s (results in Figure 5b). The infrared image was captured using a thermal imager (Uti120S, UNI-T, CN). For the grasping demonstration, the duty cycle modulation is the same as the single leg actuation. The outer shell of the gripper is 3D-printed with PETG.

### 4.5 Robotic Glove

The experiment setup for measuring the bending angle and torque output of the bending type E-FPTA in the robotic glove is shown in Figure S16. The bending angle was measured using an IM948 inertial measurement unit (IMU), and the torque output was measured using a torque sensor (0.2 N m S002 torque sensor, Tianjin STBD Technology, CN). During the two tests, the two bending actuators were stimulated using 0.5 A current with duty cycle modulation of 25 s heating, 7 s cooling, and 11 s heating.

Referring to the robotic glove design guidelines in Polygerinos’s work,^46^ the heights of all actuators in the glove should be less than 20 mm. The cross-section area of these actuators is a combination of semicircle and rectangular with an elastomer wall thickness of 2.5 mm. Parameters of the bending type E-FPTA in the robotic glove are listed in Table S3. The weight of the three-finger robotic glove is only 200 g when all E-FPTAs are fully filled with Novec 7100. All demonstration of the glove is actuated using 0.5 A current. When all actuators are stimulated simultaneously, the power input is 80 W and the duty cycle modulation is the same as the experiment setup. The experiment protocols were approved by the Human Research Ethics Committee of the University of Hong Kong with the number EA1903040. We have the informed written consent of the participant.

### 4.6 Analytical Modeling of the E-FPTA

The relationship between vapor pressure and temperature of the low boiling point fluid is given in Antoine equation, ln *P* = A – B/ (*t* + 273.15 + C), where *P* is pressure in Pascal, *t* is the temperature in Celsius degree, A, B, and C are component-specific constants. The equation parameters of Novec 7100 are given in Table S1 and the analytical pressure value is shown in Figure S3. The theoretical pressure is far larger than the measured vapor pressure in our experiments because all parameters are tested in a constant volume vessel and stable state. Waiting for the vapor–liquid phase to stabilize requires several days.^47^ Due to the different experiment conditions, like short response time and expansible rubber chamber, we corrected the Antoine equation to get an empirical equation for E-FPTA modeling:
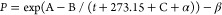
1

α is −25 °C and β is −7 Pa in this work.

When the chamber temperature rises, the pressure increases, and the chamber also expands. This process is simplified and decomposed into two steps: an isochoric process first and then an isothermal process in a closed container. The initial chamber volume *V*_1_ of the E-FTA can be calculated by
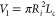
2where *R*_*i*_ and *L*_*c*_ are the radius of the inner chamber and the length of the chamber at the initial state (see Figure S5). When the chamber is filled with Novec 7100 at the beginning and the current pressure *P*_2_ can be gained from eq 1 during the isochoric process. For the isothermal process, the chamber expands to a volume of *V*_2_ = π*r*_*i*_^2^*L*_*c*_λ_*z*_, and it can be calculated by
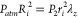
3

*P*_*atm*_ is the initial pressure equaling the pressure of the atmosphere, λ_*z*_ is the axial stretch per unit actuator length, and *r*_*i*_ is the radius of the chamber after expanding and we assume the chamber is always a standard cylinder. To solve eqs 1-3, the modeling of fiber-reinforced extending actuator is required to calculate the *R*_*i*_, *r*_*i*_, and λ_*z*_ under a constant pressure input.

The E-FPTA can be simplified into two sections: an isotropic inner layer made of pure rubber and an anisotropic flexible electrothermal composite outer layer. For the isotropic inner layer, a neo-Hooken model is selected to describe the strain energy *W*^(*in*)^:
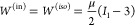
4where μ is the shear modulus of the silicone rubber. *I*_1_ represents for the first tensor invariants, and *I*_1_ = *tr*(*B*). *B* is the left Cauchy-Green tensor, and it can be calculated via the deformation gradient *F*, *B* = *FF*^*T*^. *F* denotes the extension, expansion, and twisting morphing of the soft actuator,^48^ and
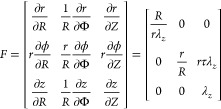
5where *R*, Φ, Z and *r*, ϕ, *z* are the radial, circumferential, and longitudinal coordinates in the initial and pressurized configurations, respectively. τ is the twisting per unit length of the soft actuator, and τ = 0 for the soft extending actuator composed of two symmetric fibers. For the soft extending actuator composed of the two sets of fibers, the strain energy of the anisotropic outer layer is
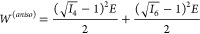
6where *E* is the Young’s modulus of metallic fibers, *I*_4_ and *I*_6_ are the fourth and sixth tensor invariants, *I*_4_ = *s*_1_. *s*_1_ and *I*_6_ = *s*_2_. *s*_2_.^29^ Fiber orientations can be calculated by *s* = *FS*, *S* = [0 cos α sin α]*^T^*is a unit vector tangent to the conductive fiber in the initial configuration of the actuator (see Figure S5).
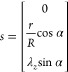
7
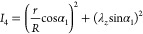
8
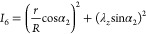
9

The strain energy of the anisotropic outer layer *W*^(*out*)^ can be calculated by
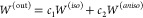
10

*c*_1_ and *c*_2_ are the volume fractions of rubber elastomer and metallic fiber,
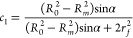
11and *c*_2_ = 1 – *c*_1_. *r*_f_ is the radius of the metallic fiber. The total strain energy of the soft actuator can be calculated by
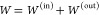
12

The Cauchy stresses σ of the two layers can be calculated using strain energies,^49^
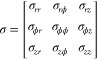
13

The Cauchy stresses of the isotropic layer σ^(*in*)^, and the anisotropic layer σ^(*out*)^ can be expressed by
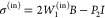
14
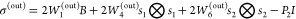
15

, *I* is the identity matrix.

The strain and radius of the extended actuator can be calculated from Cauchy equilibrium equations, axial load (*N*) balance, and axial moment (*M*) equilibrium under no load:
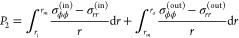
16
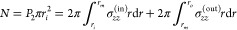
17
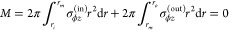
18

*r*_*o*_ is the inner radius of the anisotropic layer under pressurization.^29^

The relationship between the parameter changes of E-FPTA and chamber pressure can be calculated by eqs 4-18. λ_*z*_ and *r*_*i*_ can be found at a temperature input *t* by solving eqs 1-18. The relationship between λ_*z*_ and *P*_2_ of the baseline E-FPTA is given in Figure S17 to verify the modeling of the fiber-reinforced extending actuator.

The shear modulus for the 0 A silicone rubber used in this study is 0.028 MPa and validated via the uniaxial tensile test. Figure S18 shows the silicone rubber specimen and the results of the uniaxial tensile test. The modeling of the relationship between strain and temperature of extending type E-FPTA is compared with the experiment data in Figure 2c. The theoretical values were calculated using MATLAB.

## Supporting Information Available

Failure analysis of E-FPTA, theoretical vapor pressure of low-boiling point fluids, effect of fiber pitch in heating process, key parameters of E-FPTA, structure of the bistable TPE layer, performance and the load capability of soft gripper, actuation of robotic glove, fabrication process of E-FPTA, Joule heating of different conductive fibers, effect of the conductive fibers in E-FPTAs, experimental setups, modeling verification of fiber-reinforced soft actuator, strain stress curve of the 0 A silicone rubber, parameters of Antoine equation, parameters of specimens and soft actuators in robotic glove, comparison between the proposed E-FPTA and existing studies (PDF)
Extending type E-FPTA (MP4)
E-FPTA with programmable deformation (MP4)
Octopus-inspired soft gripper (MP4)
Soft robotic glove (MP4)
