# Supplementary material for: Embedded Conductive Fiber for Pumpless Liquid–Gas Phase Transition Soft Actuation
Source: ACS Appl Mater Interfaces. 2025 May 5;17(19):28682–92. doi: 10.1021/acsami.5c03424 (PMC12086841; doi:10.1021/acsami.5c03424)
Supplement: Supplementary file 1 — am5c03424_si_001.pdf [file am5c03424_si_001.pdf]

Supporting Information

Embedded Conductive Fiber for Pumpless Liquid-gas Phase Transition Soft Actuation

Hao Liu,[1] Changchun Wu,[1] Senyuan Lin,[1] Yunquan Li,[2] Yang Yang,[3] James Lam,[1] Ning Xi,[4] and Yonghua Chen[1,]*

[1]Hao Liu, Changchun Wu, Senyuan Lin, James Lam, and Yonghua Chen − Department of Mechanical Engineering, The University of Hong Kong, 999077, Hong Kong. E-mail: yhchen@hku.hk

[2]Yunquan Li − Shien-Ming Wu School of Intelligent Engineering, South China University of Technology, Guang Zhou, 510640, China.

[3]Yang Yang − School of Automation, Nanjing University of Information Science and Technology, 210044, Nanjing, China.

[4]Ning Xi − Department of Data and Systems Engineering, The University of Hong Kong, 999077, Hong Kong.

*Corresponding authors. E-mail addresses: yhchen@hku.hk

This file includes:

Figs. S1 to S18

Tables S1 to S5

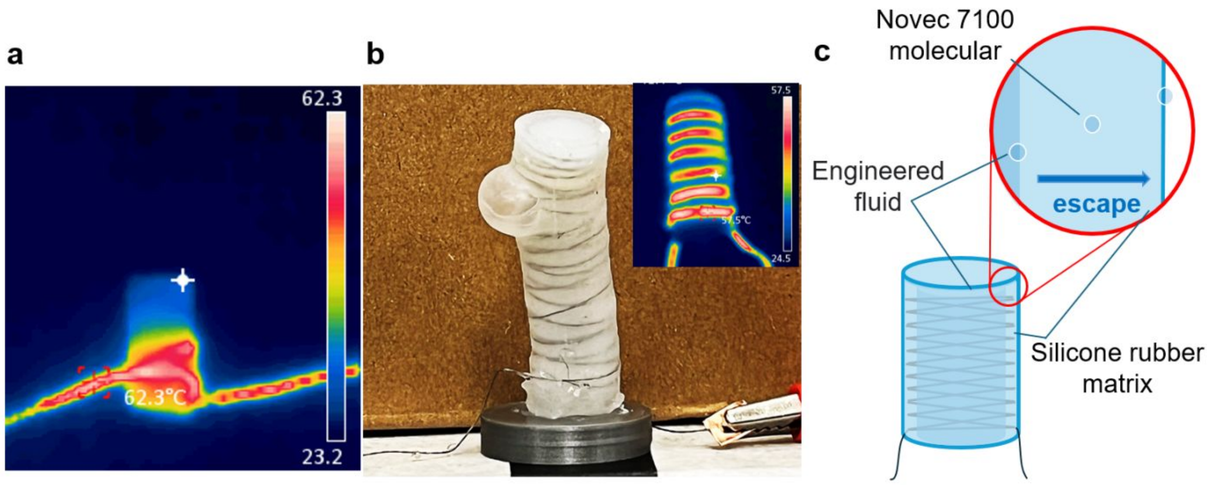

Figure S1. Common failure types of E-FPTA. (a) Uneven heating due to short circuit. (b) Silicone rubber explosion caused by thermal degradation and inhomogeneous heating. (c) Leakage of the engineered fluid.

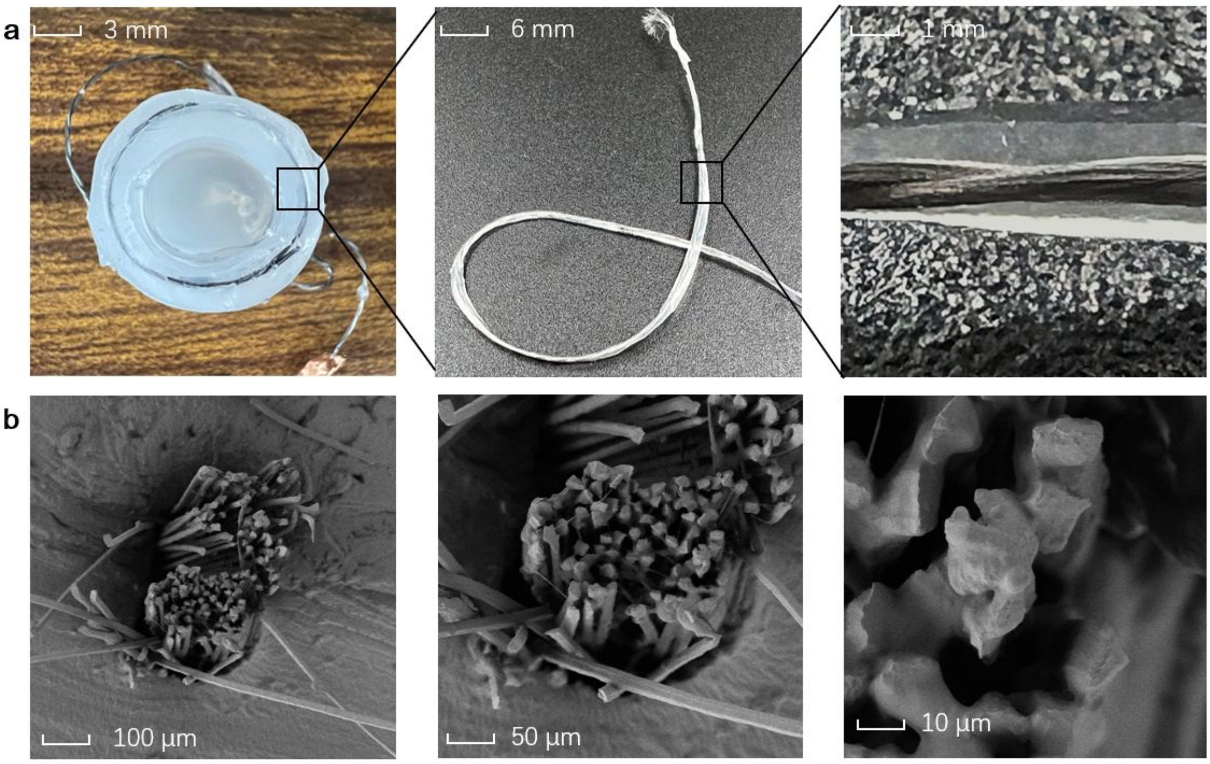

Figure S2. Materials of the E-FPTA. (a) Conductive steel fiber coated by a silicone rubber. The steel fiber is a two-ply helical yarn comprising torsionally coupled steel microfibers, and the average diameter of the steel microfiber is 12 µm. (b) SEM characterizations of the steel fiber coated by a silicone rubber.

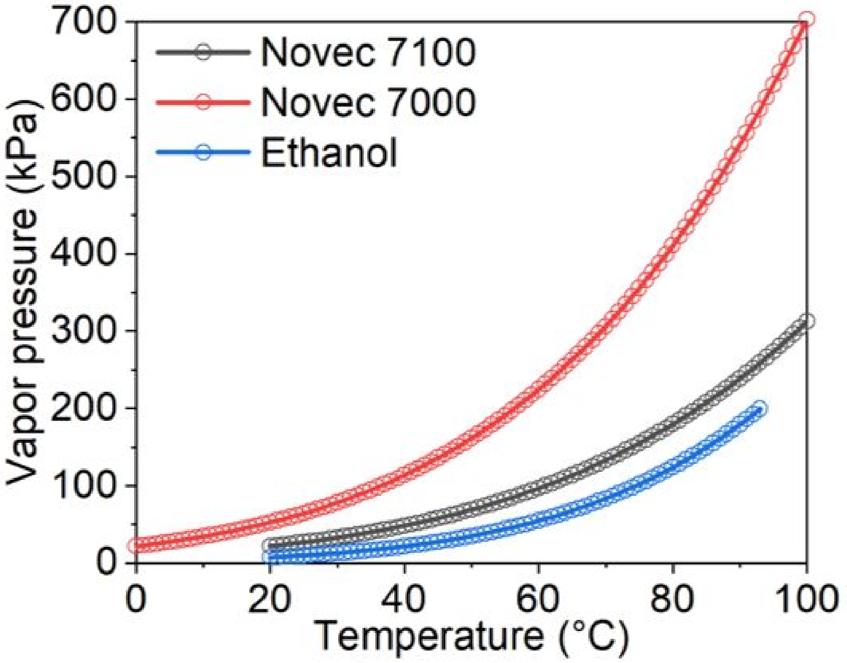

Figure S3. Theoretical vapor pressure of Novec 7100, Novec 7000, and ethanol based on Antoine equation.

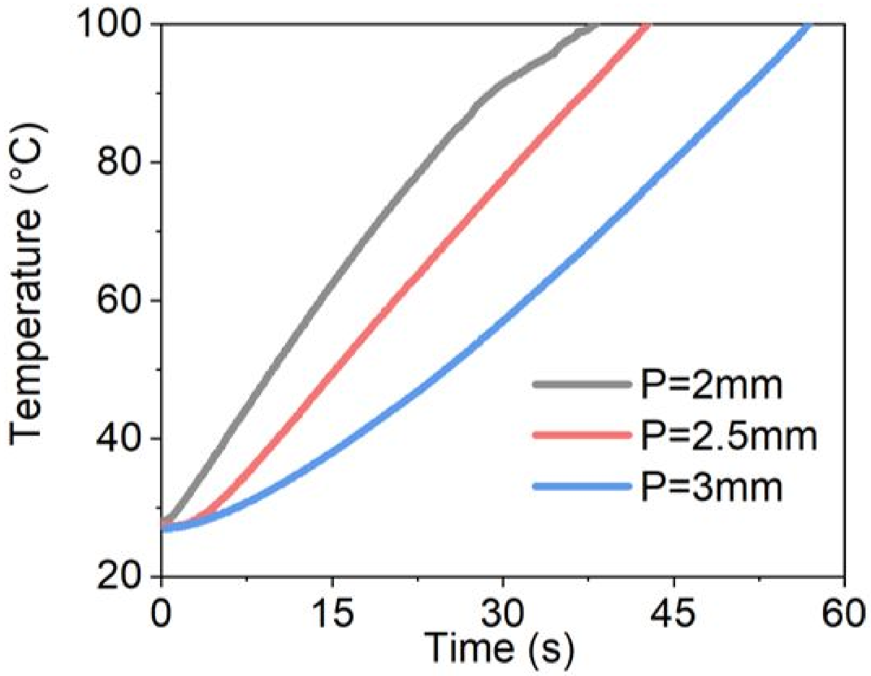

Figure S4. Temperature increase of the E-FPTA with different fiber pitch.

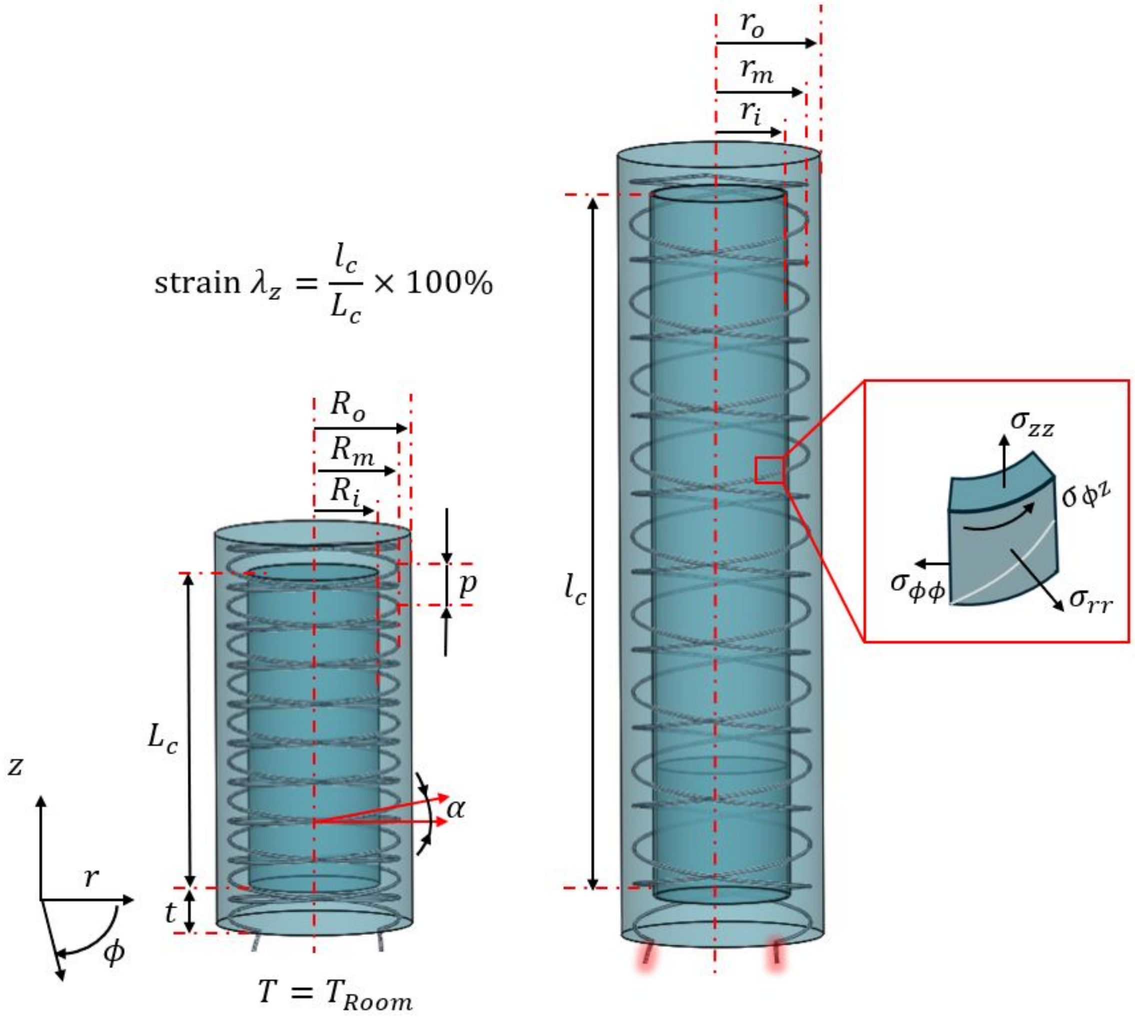

Figure S5. Parameters of the E-FPTA.

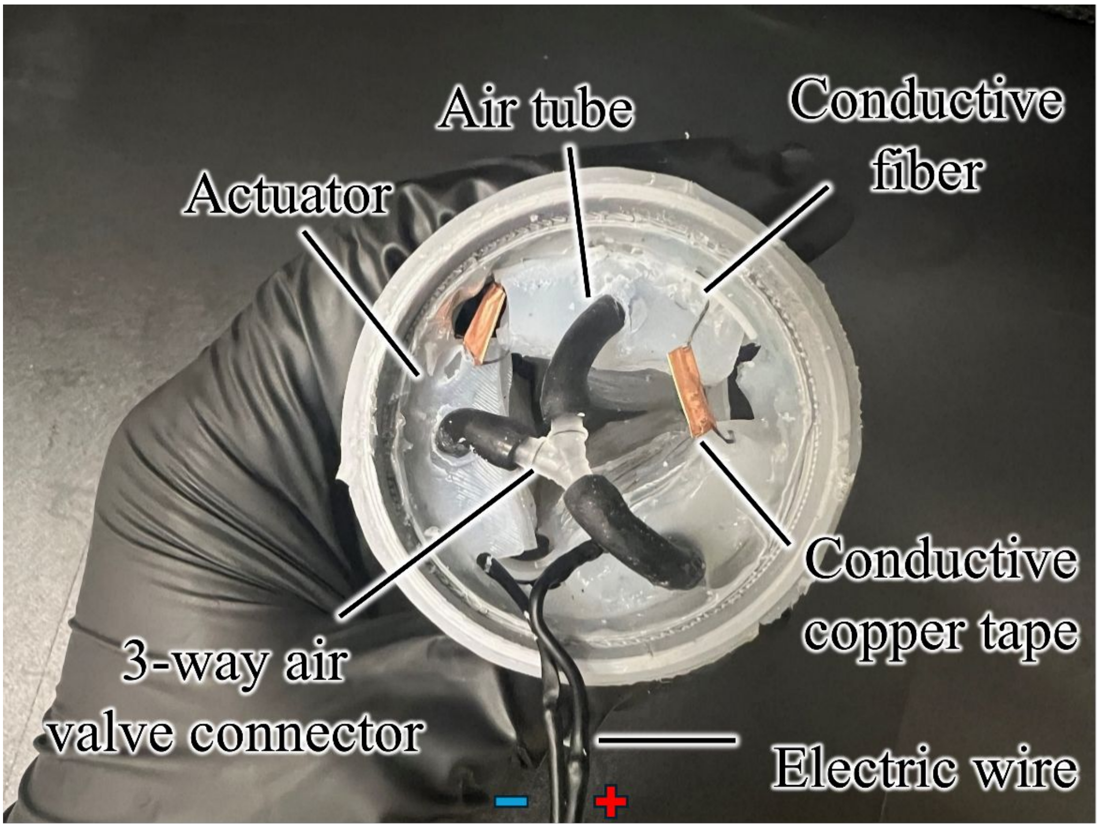

Figure S6. The inner structure of the soft gripper.

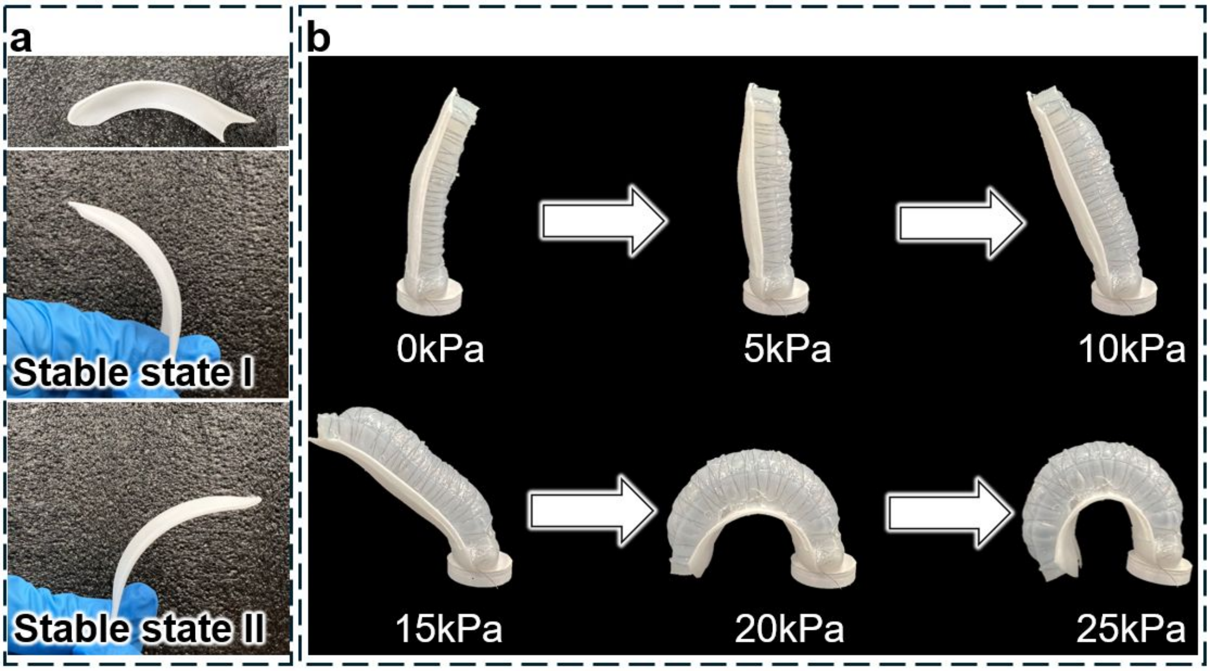

Figure S7. Actuator leg with a bistable TPE layer. (a) 3D-printed leaf-like bistable TPE layer. The TPE layer can reach two stable states for grasping stability. (b) Bending angle of the single actuator leg when increasing the pressure from 0 kPa to 25 kPa.

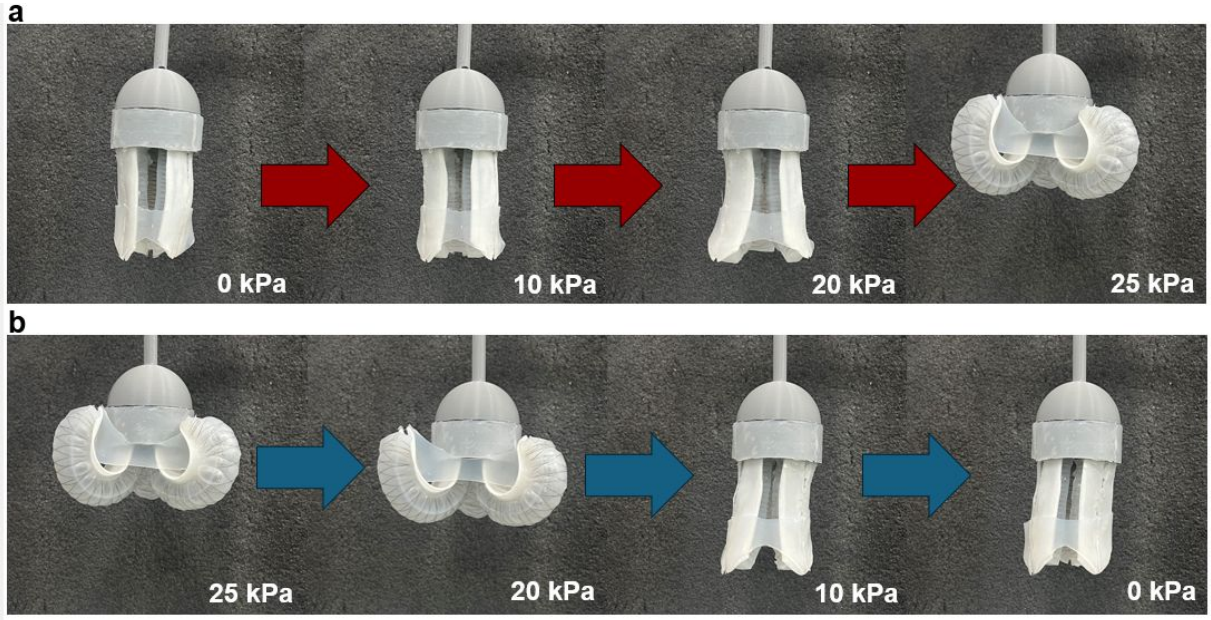

Figure S8. Soft gripper under different pressure inputs. (a) Increasing the pressure from 0 kPa to 25 kPa. Benefiting from the bistable layer, the actuator legs keep closed when the chamber pressure is below 20 kPa allowing a stable grasping for small and stick-like objects. (b) Decreasing the pressure from 25 kPa to 0 kPa. Different from the actuation process, the gripper's legs remain open at the 20 kPa chamber pressure in the recovery process, so the operator has more time to align the gripper with the grasped objects.

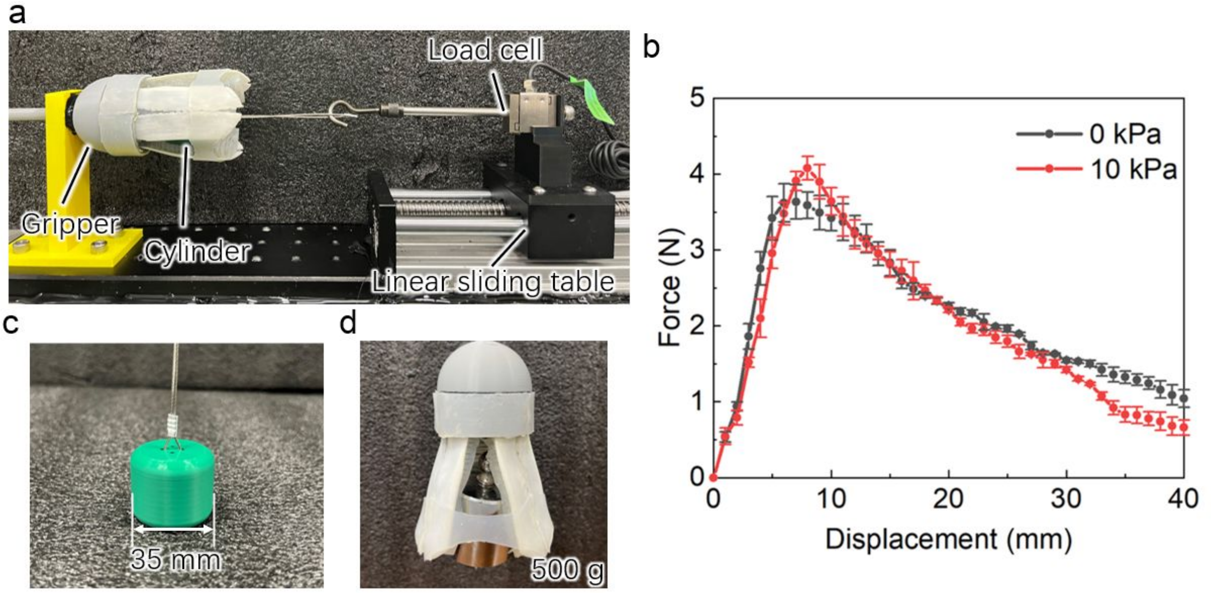

Figure S9. Load capability of the soft gripper. (a) Experiment setup of the pull-out force test. (b) Relationship between the force output of the soft gripper and the displacement of the objects. Compared to the 0 kPa situation, the soft gripper under 10 kPa can output a large force at the initial displacement due to the elastomer inflation but also leads to a small force when the object is almost pulled out because the ends of the actuators bend to reduce interaction. (c) 3D-printed PLA cylinder used in the load capability test. (d) Lifting a 500 g weight with a diameter of 44 mm.

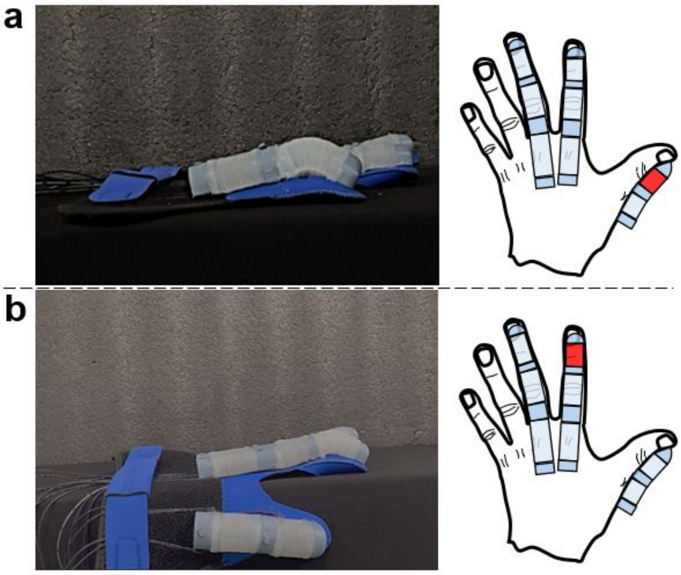

Figure S10. Single joint actuation of the robotic glove. (a) Bending the IP joint in the thumb. (b) Bending the DIP joint in the index finger.

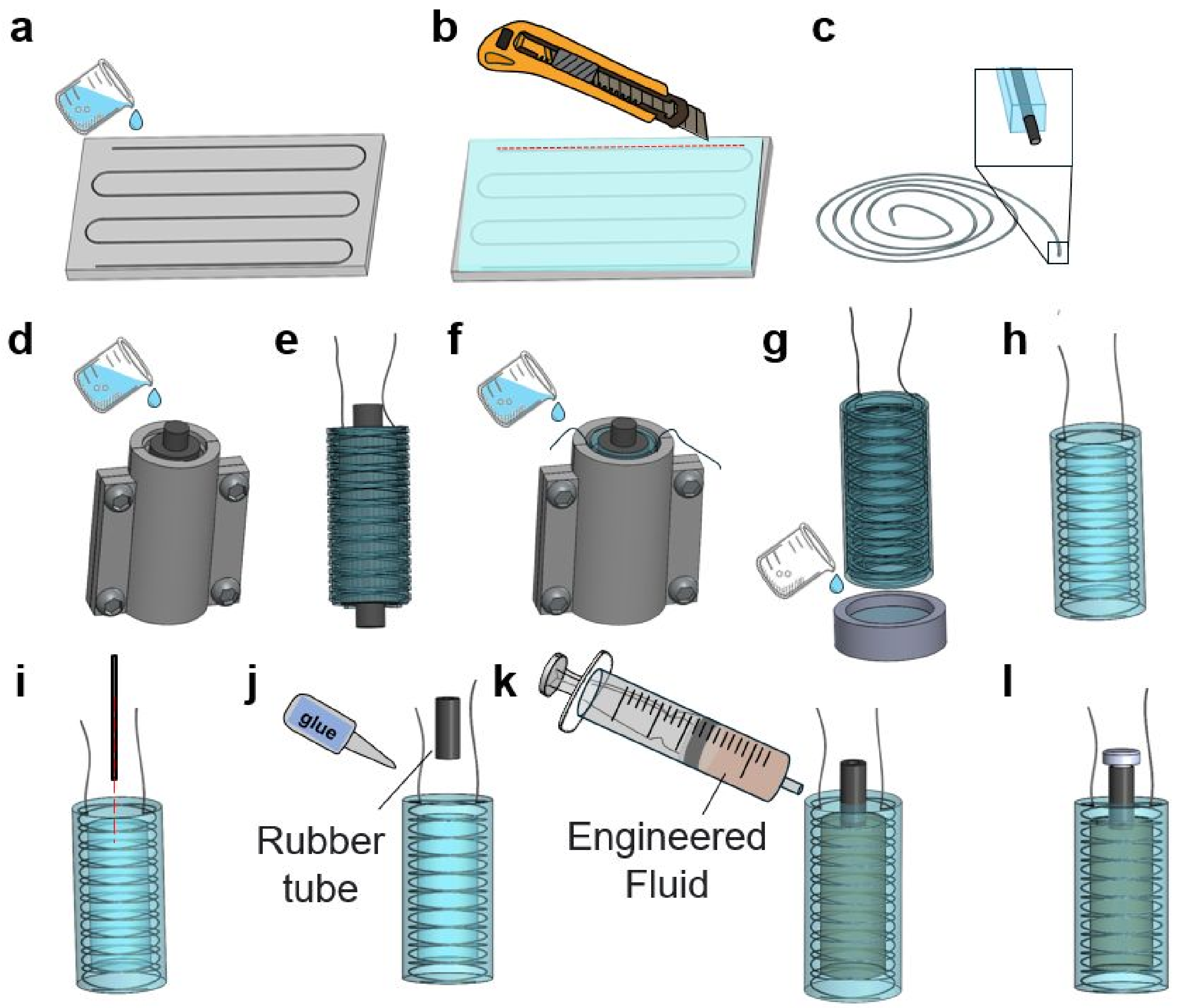

Figure S11. Fabrication process of the E-FPTA. (a) Putting a conductive fiber on a plate and pouring uncured rubber on it. (b) Cutting the rubber along the fiber. (c) Separating the conductive fiber from the rubber layer and getting a flexible electrothermal composite fiber. (d) Pouring the uncured rubber into a mold. (e) Winding the flexible electrothermal composite fiber on the mold stripped from step d. (f) Pouring the uncured rubber into a mold inserted with part from the previous step. (g-h) Sealting two ends of the hollow chamber from the previous step. (i) Puncturing a hole in the bottom of the actuator. (j) Inserting a rubber tube into the hole, and sticking it using glue. (k) Injecting low bowling point liquid into the chamber. (l) Sealing the rubber tube using a plug.

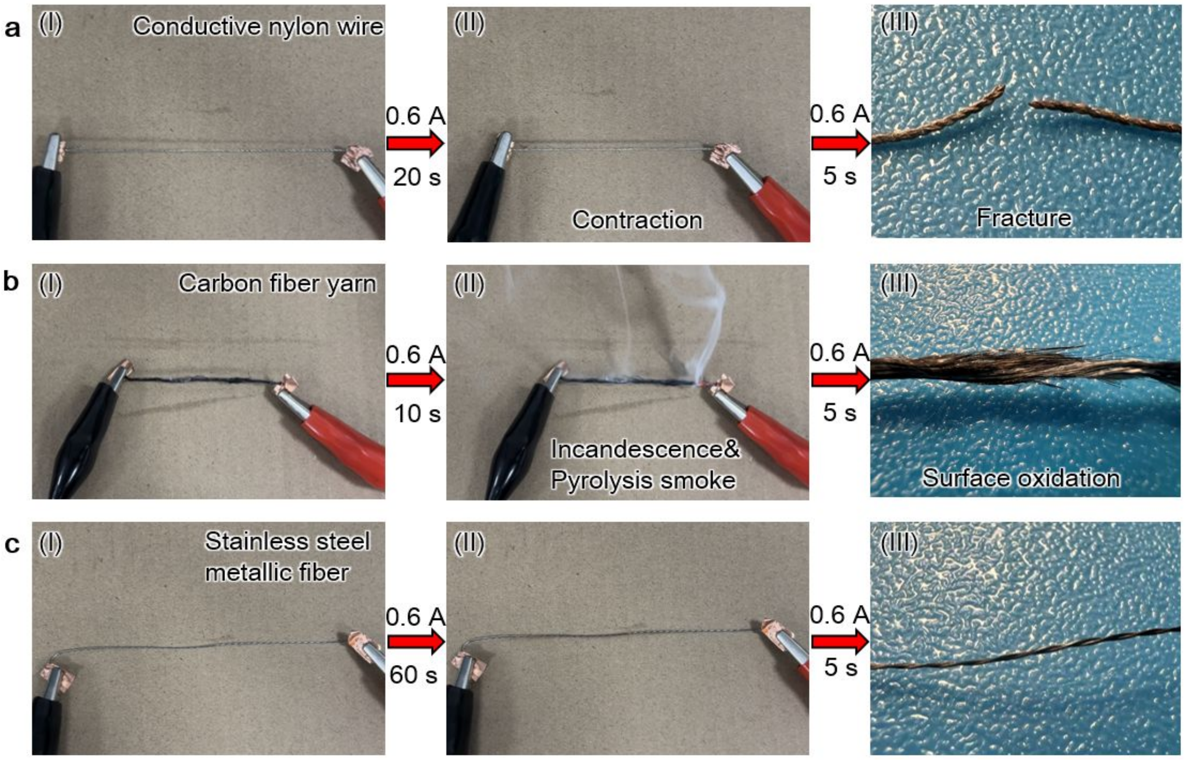

Figure S12. Joule heating of different conductive fibers. (a) Conductive nylon wire. Conductive nylon wire shrinks during heating and breaks when the temperature is higher than 60 °C. (b) Carbon fiber yarn. Carbon fiber yarns produce incandescence and pyrolysis smoke under Joule heating. (c) Stainless steel metallic fiber. The steel fiber shows high mechanical strength and chemical stabilization. (I) Fibers under the initial state. (II) Fibers heated by electricity. (III) Fibers cooling to the room temperature.

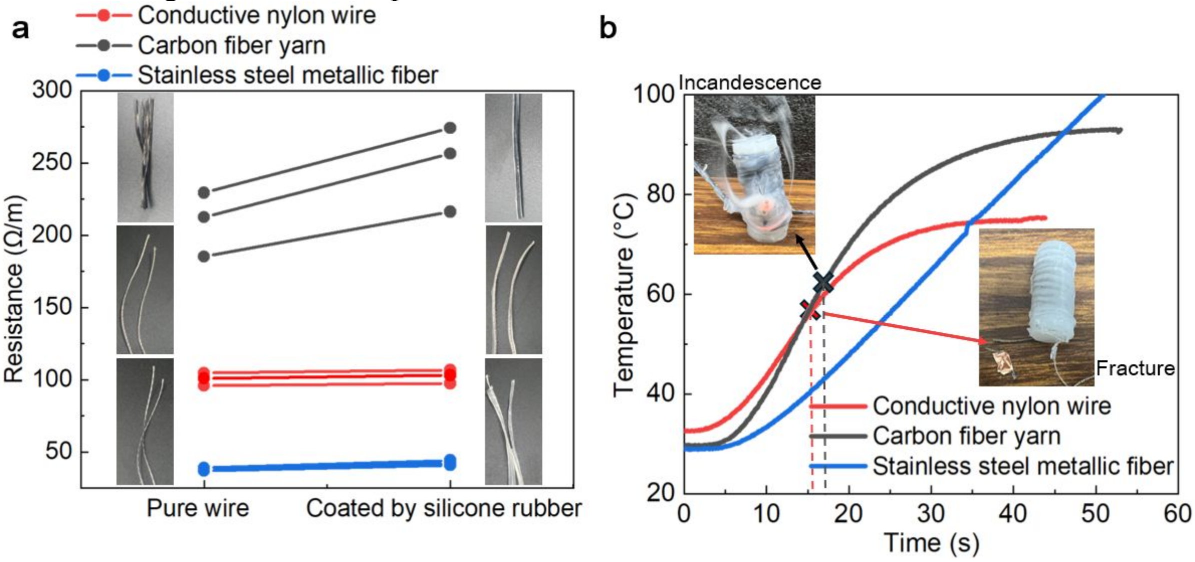

Figure S13. Effect of the conductive fibers in E-FPTA. (a) Resistance of the conductive nylon wire, carbon fiber yarn, and stainless steel metallic wire after sealed in silicone rubber. The resistance of the carbon fiber yarn was increased from 209.1 ± 18.1 Ω/m to 249.0 ± 24.2 Ω/m due to carbon fibers isolated by the silicone rubber matrix leading to the short circuit. (b) E-FPTAs composed of different fibers heating under 0.6A current. E-FPTAs composed of the carbon fiber yarn and conductive nylon wire show a high heating speed due to high power input (104.5 W and 26.4 W, respectively), but they failed quickly because of weak physical stability.

After the failure of E-FPTAs, the temperature in the chamber still rises due to the delay in heat transfer.

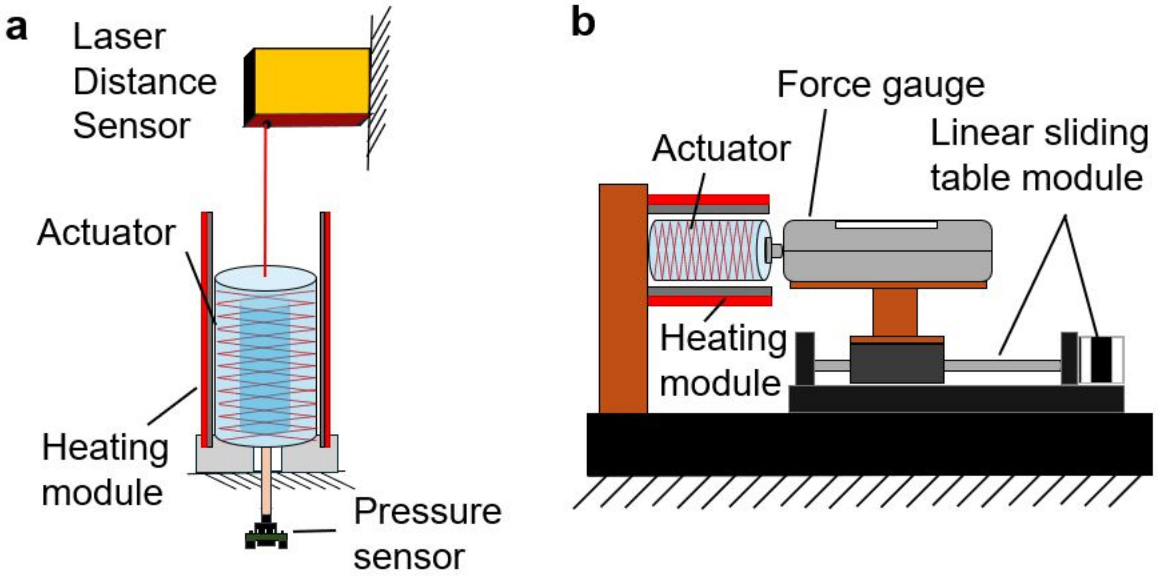

Figure S14. Experimental setups for the thermodynamic tests of the E-FPTA. (a) Measuring the strain of the E-FPTA at constant temperatures. (b) Measuring the relationship between strain and force output at constant temperatures.

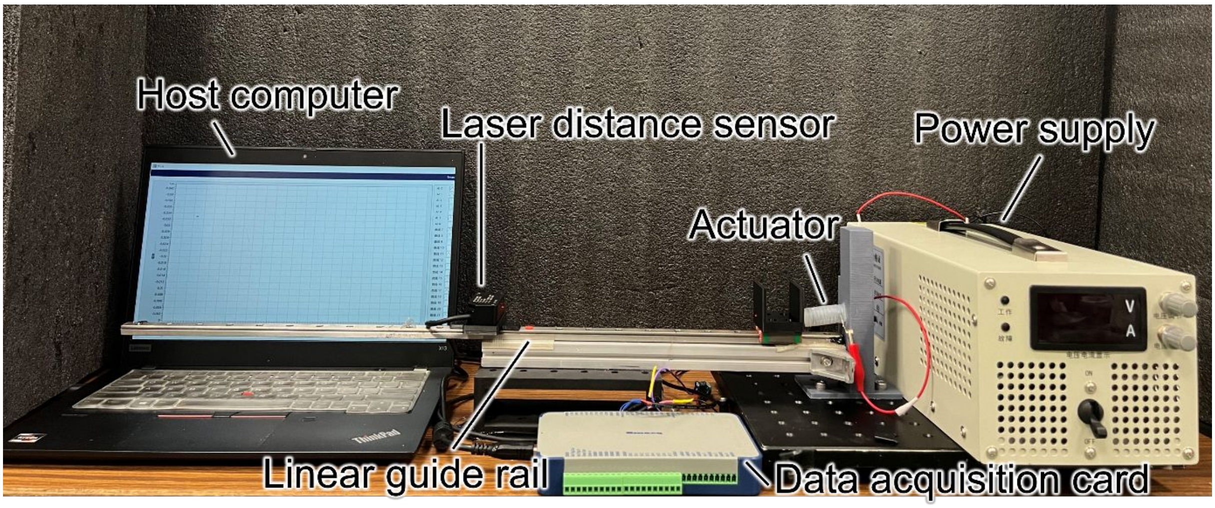

Figure S15. Test apparatus for dynamic test of the E-FPTA.

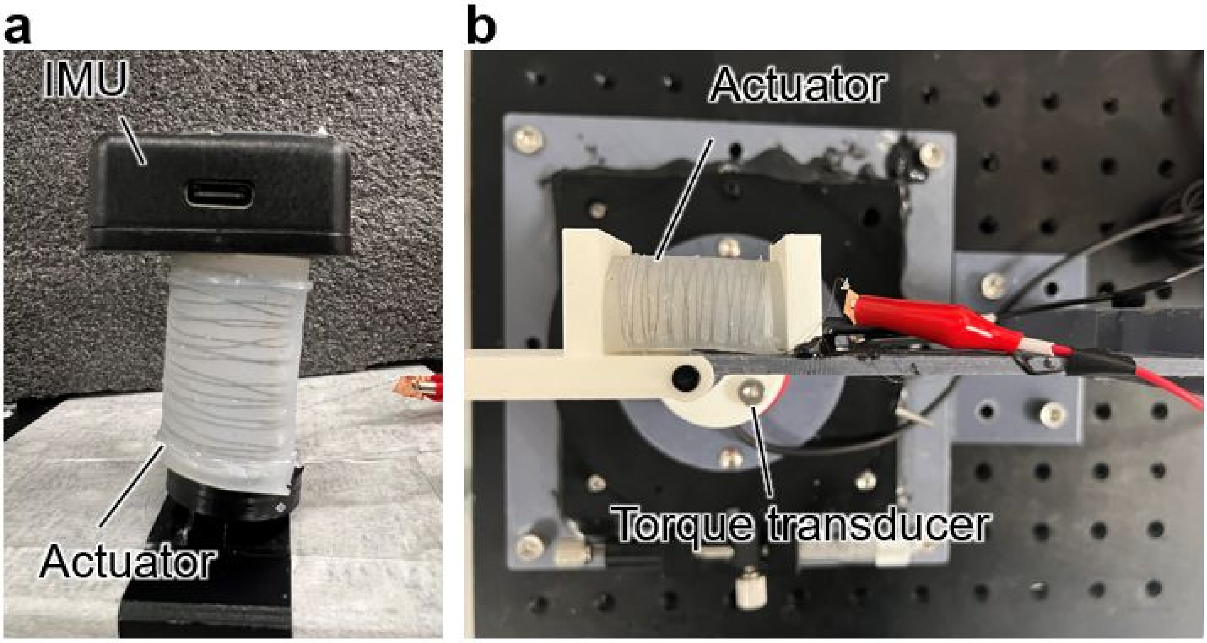

Figure S16. Test setup of the bending actuator. A) Measuring the bending angle. B) Measuring the torque output.

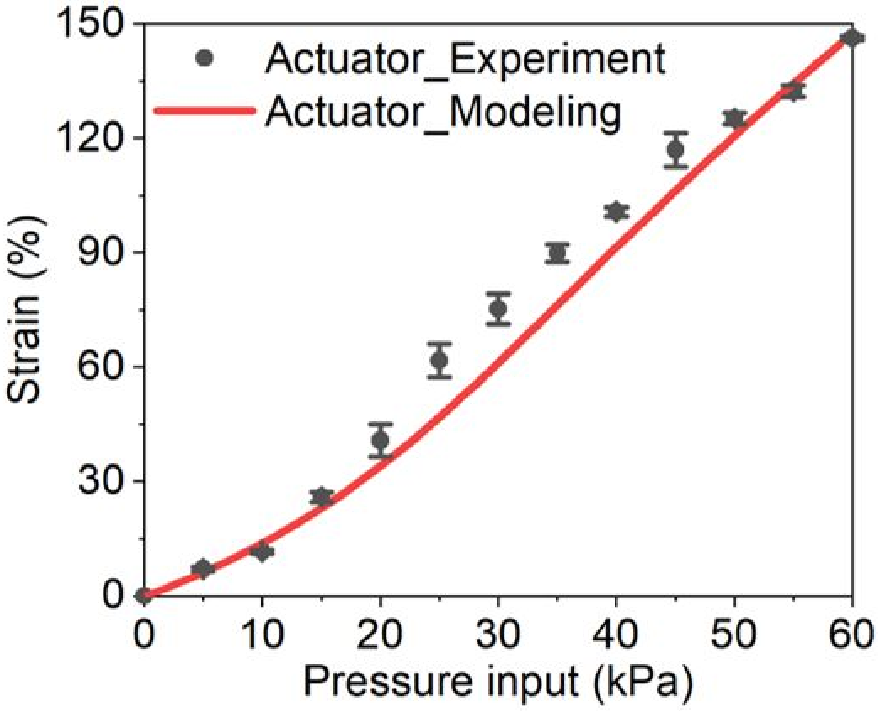

Figure S17. Modeling validation of fiber-reinforced extending actuator made of 0A silicone rubber.

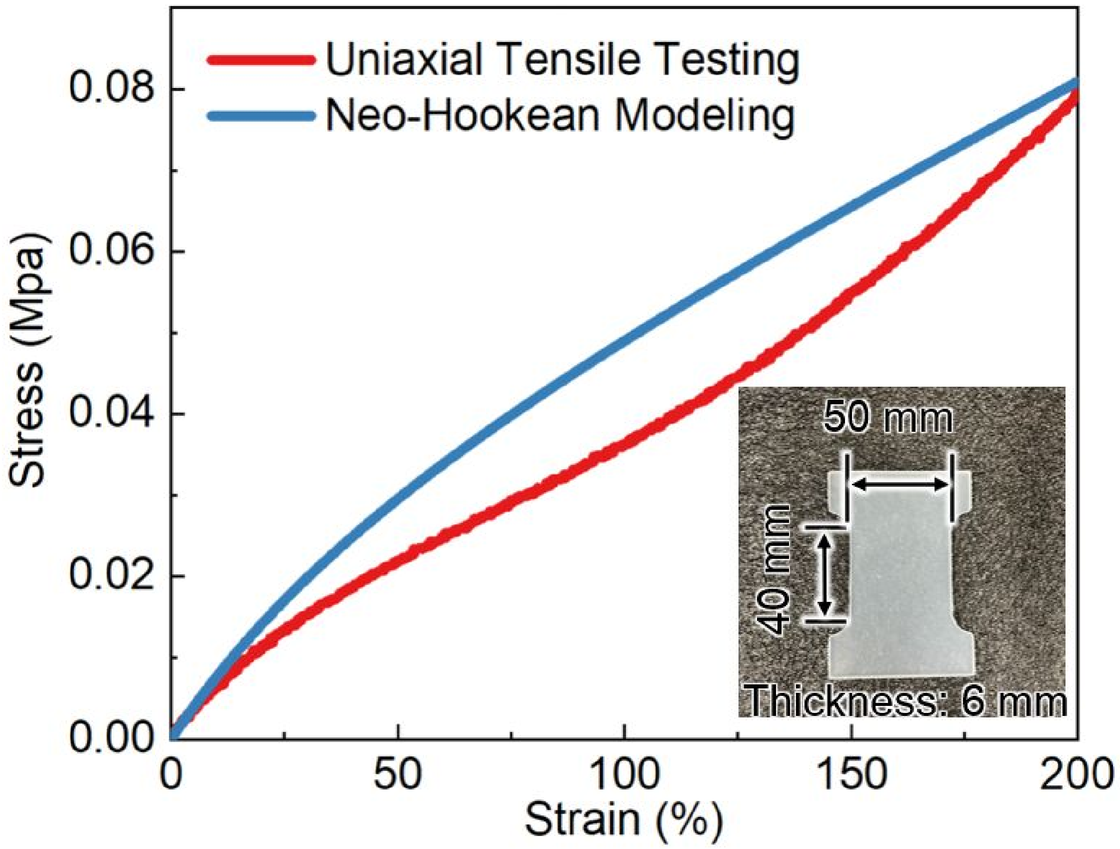

Figure S18. Strain stress curve of the silicone rubber with hardness Shore 0A. The theoretical stress σ is calculated by the Neo-Hookean model, $\sigma = \mu\,(\lambda - \lambda^{-2})$, where μ is the shear modulus of the silicone rubber and λ is the axial stretch ratio of the silicone rubber specimen.

Supplementary Table

Table S1. Antoine equation parameters of the Novec 7100, Novec 7000, and Ethanol.

Fluid	A	B	C
Novec 7100	22.415	3641.9	0
Novec 7000	22.978	3548.6	0
Ethanol	23.594	3681.1	-46.424

Antoine equation: $\ln P = A - B/(t + 273.15 + C)$; P is the vapor pressure in Pascal and t is the temperature in Celsius degrees.

Table S2. Design parameters of the actuator specimens.

No.	Hardness (Shore A)	P (mm)	l_c (mm)	r_m (mm)	r_i (mm)	r_o (mm)
1(baseline)	0	3	30	6.25	5	7.5
2	10	3	30	6.25	5	7.5
3	20	3	30	6.25	5	7.5
4	30	3	30	6.25	5	7.5
5	0	2	30	6.25	5	7.5
6	0	2.5	30	6.25	5	7.5
7	0	3	30	6.25	5	7.5
8	0	3	30	8.5	7	9.5
Contracting	0	NA	30	6.25	5	7.5
Twisting	0	3	30	6.25	5	7.5
Bending	0	3	60	6.25	5	7.5
Helical bending	0	3 & 60	60	6.25	5	7.5

Table S3. Design parameters of the bending E-FPTA in robotic glove.

Finger	Joint	Outer Radius (mm)	Length (mm)	Hardness (Shore A)	Resistance (Ω)	Wall thickness (mm)
Middle & Index fingers	MCP	10	30	0	53	2.5
	PIP	10	20	0	35	2.5
	DIP	10	16	0	28	2.5
Thumb	MCP	13	27	0	41	2.5
	IP	13	21	0	32	2.5

Table S4. Comparison between the E-FPTA and existing phase change actuators.

	Actuator	Mechanism	Response time (s)	Pressure (kPa)	Power input (W)
This work	E-FPTA	Joule heating	50	60	12-17
Han et al. [35]	Fiber-reinforced actuator	Photothermal effect	400	Not given	Not given
Hao et al. [37]	McKibben muscle	Acoustothermal effect	30	8.16	Not given
Mirvakili et al. [39]	McKibben muscle	Magnetothermal effect	10	2.1	100
Yoon et al. [40]	Soft Thermo-pneumatic actuating module	Thermoelectric effect	58	Not given	14.8
Chellattoan et al. [41]	Pneu-net actuator	Joule heating	54	15	30

Table S5. Comparison between the E-FPTA and EAPs.

EAPs	Mechanism	Deformation capability	Relative speed*	Size (mm^3)	Driving voltage (MVm^{-1})
E-FPTA	Electrothermal liquid-gas phase transition	Strain 120 %	Medium	15*15*30	0.93
DEA [50]	Maxwell stress	Strain 253 %	Fast	0.5*60*30	46
IPMC [51]	Electromechanical ion migration	Bending strain 0.48 %	Medium	0.025*4*35	4×10^{-5}
Electroactive hydrogel (EAH) [52]	Electromechanical ion migration	Strain 85 %	Slow	0.01*0.045*0.9	2.2×10^{-2}
Conductive SMP composite [53]	Electrothermal shape memory effect	Strain 8.1 %	Fast	0.04*3*3	40
Piezoelectric polymers [54]	Piezoelectric effect	Bending strain 0.06 %	Fast	0.025*5*25	50

*Relative speed: Fast means the response speed < 1 s; Medium means the response speed less than 60s; Slow means the response speed larger than 1 min. The response speed of EAPs is related to the actuator size and power supply.
